# Supplementary material for: Broad-spectrum CRISPR-Cas13d-mediated strategy for combating human coronaviruses
Source: Mol Ther Nucleic Acids. 2026 Mar 6;37(2):102888. doi: 10.1016/j.omtn.2026.102888 (PMC13126349; doi:10.1016/j.omtn.2026.102888)
Supplement: Document S1. Figure S1 and Tables S1–S3 [file mmc1.pdf]

OMTN, Volume *37*

Supplemental information

Broad-spectrum CRISPR-Cas13d-mediated strategy for combating human coronaviruses

Zhenghao Yu, Mouraya Hussein, Yuanling Bao, Ana Alcalá-Lalinde, Pascal Zion Kroon, Eva Thuillier, Zhi Zhang, Luis Enjuanes, Sonia Zuñiga, Ben Berkhout, and Elena Herrera-Carrillo

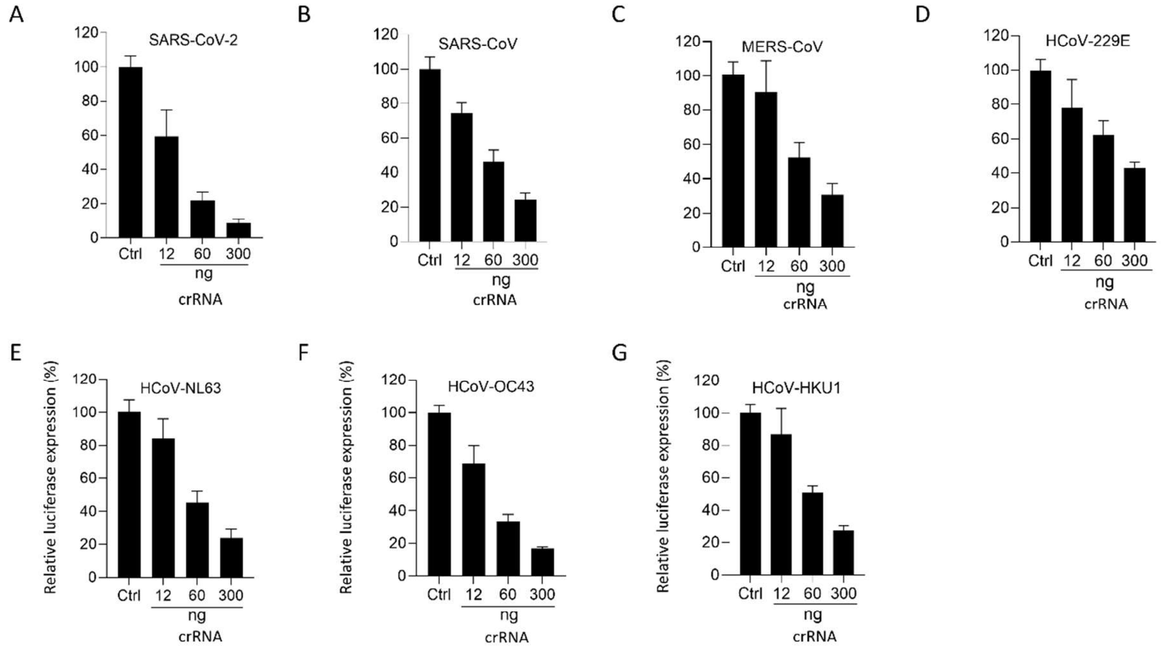

Figure S1. Dose-dependent inhibition of human coronaviruses by CRISPR-Cas13d/crRNA. The effect of Cas13d/crRNA2 dose on viral gene expression was evaluated using three transfection doses (12 ng, 60 ng, and 300 ng). After two days, luciferase levels were measured to assess the inhibition efficiency. A non-targeting crRNA (Ctrl), which does not target any human coronavirus, served as a negative control, with its luciferase expression normalized to 100%. Luciferase levels at different transfection doses are shown as percentages relative to the control (Ctrl).

Table S1. List of sequences inserted into the luciferase reporter

Human coronavirus	Sequence(5’-3’)
SARS-CoV-2	gtttatagtgatgtagaaaaccctcaccttATGGGTTGGGATTATCCTAAATGTGAtagagccatgcctaacatgcttagaattat
SARS-CoV	gtttacagtgatgtagaaactccacaccttATGGGTTGGGATTATCCAAAATGTGAcagagccatgcctaacatgcttaggataat
MERS-CoV	ttgtataaagatgttgataatccgcatcttATGGGTTGGGATTACCCTAAGTGTGAtagagctatgcctaatatgtgtagaatctt
HCoV-229E	ctgatggccgatgttgacgatcctaaattgATGGGATGGGACTATCCTAAGTGTGAtagagctatgccctcaatgattcgtatgtt
HCoV-NL63	ttaatcgatggtgttgaaaaccctatgctcATGGGTTGGGATTATCCCAAATGTGAtagagctttgcctaacatgatacgtatgat
HCoV-OC43	cttattaaagatgtcgacaatcctgtacttATGGGTTGGGATTATCCTAAGTGTGAtcgtgctatgccaaacatactacgtattgt
HCoV-HKU1	cttataaaggatgttgacaaccctgttcttATGGGTTGGGATTATCCTAAATGTGAtcgtgctatgccaaatattttgcgtattgt

Uppercase letters in the sequences indicate the target regions of each human coronavirus, while lowercase letters represent the 30-nucleotide flanking regions derived from the corresponding viral genome.

Table S2. Primers and probes used for qPCR and detection of escape virus.

Name	Sequence (5'-3')
SARS-CoV-2-sgmRNA-Forward	CCAACCAACTTTCGATCTCTTGT
SARS-CoV-2-sgmRNA-Reverse	GGGTGCATTTCGCTGATTTT
SARS-CoV-2-sgmRNA-Probe	TTCTCTAAACGAACAAACTA
SARS-CoV-sgmRNA-Forward	GCCAACCAACCTCGATCTCTTGT
SARS-CoV-sgmRNA-Reverse	AATCTGTGGGTCCACCAAATGTA
SARS-CoV-sgmRNA-Probe	CTCTAAACGAACAAATTA
MERS-CoV-sgmRNA-Forward	CTTCCCCTCGTTCTCTTGCA
MERS-CoV-sgmRNA-Reverse	TCATTGTTATCGGCAAAGGAAA
MERS-CoV-sgmRNA-Probe	CTTTGATTTTAACGAATCTC
HCov-229E-sgmRNA-Forward	CAGATAGAAAAGTTGCTTTTTAGAC
HCov-229E-sgmRNA-Reverse	CAGATGCATCAGCCCATTTGAC
HCov-229E-sgmRNA-Probe	TACTTTTCTAAACTGAACGA
HCov-NL63-sgmRNA-Forward	ATAGATAGAGAATTTTCTTATTTAGAC
HCov-NL63-sgmRNA-Reverse	AATTTCTTCCTAGCAGCTCTGT
HCov-NL63-sgmRNA-Probe	ACTAAACAAAATGGCTAGTG
HCov-229E-gRNA-Forward	GAACCTGATGGCCGATGTTG
HCov-229E-gRNA-Reverse	GCTTAGAACCTAAAATCATAGCC
HCov-229E-gRNA-Probe	AAATTGATGGGATGGGACTATCCTAAGTG
HCov-NL63-gRNA-Forward	GTTGCGTACTTTAATTGATGGTGT
HCov-NL63-gRNA-Reverse	AGAGCCCAACACCATGGCTG
HCov-NL63-gRNA-Probe	CTTATGGGTTGGGATTATCCCAAATGT
18s-Forward	CGGCTACCACATCCAAGGAA
18s-Reverse	GCTGGAATTACCGCGGCT
18s-Probe	TGCTGGCACCAGACTTGCCCTC
HCoV-229E-escape-Forward	GGAACGTGCACGTACAGTGGG
HCoV-229E-escape-Reverse	GGCGTAGGCTGTAGTTGCATC
HCoV-NL63-escape-Forward	CACGCAATGCCACTGTTGTT
HCoV-NL63-escape-Reverse	GCGTCACCAGAAGTCGTACC

Table S3. Primers used for the production of crRNAs.

Name	Sequence (5'-3')
crRNA2-F	TAATACGACTCACTATAGGGaacccctaccaactggtcggggtttgaaacCACATTTAGGATAATCCCAACCC
crRNA2-R	GGGTTGGGATTATCCTAAATGTGgtttcaaaccccgaccagttggtaggggttCCCTATAGTGAGTCGTATTA
crRNA3-F	TAATACGACTCACTATAGGGaacccctaccaactggtcggggtttgaaacACATTTAGGATAATCCCAACCCA
crRNA3-R	TGGGTTGGGATTATCCTAAATGTgtttcaaaccccgaccagttggtaggggttCCCTATAGTGAGTCGTATTA
