# Supplementary material for: Broad-spectrum CRISPR-Cas13d-mediated strategy for combating human coronaviruses
Source: Mol Ther Nucleic Acids. 2026 Mar 6;37(2):102888. doi: 10.1016/j.omtn.2026.102888 (PMC13126349; doi:10.1016/j.omtn.2026.102888)
Supplement: Document S2. Article plus supplemental information [file mmc3.pdf]

Molecular Therapy
Nucleic Acids
Original Article

Broad-spectrum CRISPR-Cas13d-mediated strategy for combating human coronaviruses

Zhenghao Yu,[1,2,6] Mouraya Hussein,[1,2,6] Yuanling Bao,[1,2,6] Ana Alcalá-Lalinde,[1,2] Pascal Zion Kroon,[1,2] Eva Thuillier,[1,2] Zhi Zhang,[3] Luis Enjuanes,[4] Sonia Zuñiga,[4] Ben Berkhout,[1,2] and Elena Herrera-Carrillo[1,2,5]

[1]Amsterdam UMC, University of Amsterdam, Medical Microbiology and Infection Prevention, Meibergdreef 9, Amsterdam, the Netherlands; [2]Amsterdam Institute for Immunology and Infectious Diseases, Amsterdam, the Netherlands; [3]Institute for Logic, Language and Computation (ILLC), University of Amsterdam, Amsterdam, the Netherlands; [4]Department of Molecular and Cell Biology, National Center of Biotechnology (CNB-CSIC), Darwin 3, Madrid, Spain; [5]Institute of Parasitology and Biomedicine "López-Neyra" (IPBLN), Spanish National Research Council (CSIC), Avda. del Conocimiento 17. P. T. Ciencias de la Salud, Granada, Spain

RNA viruses evolve rapidly, enabling them to evade host immunity and antiviral therapies and complicating durable diagnostics strategies. There is a pressing need for approaches that provide broad-spectrum viral suppression and detection. We designed four Cas13d crRNAs targeting a conserved 26-nucleotide sequence in coronavirus (CoV) nsp12. Their antiviral efficacy was evaluated *in vitro* against the target sequences of all seven human CoVs, showing potent activity across the tested samples. The same crRNAs were adapted for a Cas13d-based specific high-sensitivity enzymatic reporter unlocking (SHERLOCK) assay to detect multiple human CoVs, demonstrating high sensitivity, with the ability to detect as few as a single copy of SARS-CoV-2 RNA, while showing no detectable signal for other seasonal respiratory viruses, such as influenza. This dual-function approach underlines the versatility and potential of CRISPR technologies in both managing and detecting viral infections. Additionally, bioinformatic analysis revealed that the crRNA targets are highly conserved across animal coronaviruses, suggesting that targeting of this sequence could facilitate the rapid development of treatment options and diagnostics during a new pandemic of an emerging coronavirus. This could significantly aid in pandemic preparedness and response efforts.

INTRODUCTION

Coronaviruses are single-stranded, positive-sense RNA viruses. They are among the largest known RNA viruses, with genomes ranging from 26 to 33 kilobases (kb).[1,2] There are seven known human coronaviruses (HCoVs): the mildly pathogenic viruses HCoV-229E, HCoV-OC43, HCoV-NL63, and HCoV-HKU1 and the highly pathogenic viruses severe acute respiratory syndrome coronavirus (SARS-CoV), Middle East respiratory syndrome coronavirus (MERS-CoV), and SARS-CoV-2. HCoV-229E, HCoV-OC43, HCoV-NL63, and HCoV-HKU1 typically cause mild respiratory symptoms similar to the common cold and were identified in 1966, 1967, 2004, and 2005, respectively.[3–6] However, these HCoVs can occasionally cause more severe diseases, including pneumonia.[7,8] And in a few cases, healthy adults infected with HCoV-229E developed acute respiratory syndrome.[9] Frequent mutations in HCoV-NL63 can help the virus escape recognition by the host immune system, which may increase the risk of severe respiratory disease in humans.[10,11] MERS-CoV, discovered in 2012, is capable of rapidly inducing symptoms, such as fever, cough, and shortness of breath, which can progress to pneumonia and respiratory distress, often resulting in death.[12] SARS-CoV, identified in 2003, and SARS-CoV-2, identified in 2019, are both capable of causing severe acute respiratory syndrome, with potentially fatal outcomes.[13–15] The zoonotic SARS-CoV-2 virus recently caused the COVID-19 pandemic, posing a significant threat to public health.[16,17] There are multiple examples of coronaviruses originating from cross-species transmission to humans from diverse animals, including bats, bovine, and mice.[18–20] Coronaviruses have an RNA genome prone to mutations due to the error-prone activity of RNA-dependent RNA polymerase (RdRp). Although the polymerase of coronaviruses possesses a unique proofreading mechanism involving an exoribonuclease domain connected to the polymerase, mutations still occur, driving virus evolution through replication-associated changes that enable adaptation. Coronaviruses have a broad host range, which may contribute to the emergence of zoonotic diseases.[21,22] This adaptability underscores the growing risk of cross-species transmission, as occurred with SARS-CoV, MERS-CoV, and SARS-CoV-2, which likely originated from bats.[18,23] The increased risk of animal-to-human spillover raises concerns about future pandemics. To better prepare for the next coronavirus outbreak, there is an urgent need to develop universal vaccines or antiviral therapies.[24,25] Broad-spectrum antiviral strategies could potentially suppress not only circulating coronaviruses but also emerging strains.

Clustered regularly interspaced short palindromic repeats (CRISPR)-Cas technology provides unprecedented genome-editing capabilities and has been extensively applied in antiviral research.[26–30] CRISPR-Cas endonucleases are classified based on various characteristics,

Received 16 September 2025; accepted 3 March 2026;
https://doi.org/10.1016/j.omtn.2026.102888.

[6]These authors contributed equally

Correspondence: Institute of Parasitology and Biomedicine "López-Neyra" (IPBLN), Spanish National Research Council (CSIC), Avda. del Conocimiento 17. P. T. Ciencias de la Salud, Granada, Spain.
E-mail: elena.herrera@ipb.csic.es

© 2026 The Authors.
Published by Elsevier Inc. on behalf of The American Society of Gene and Cell Therapy.
This is an open access article under the CC BY license (http://creativecommons.org/licenses/by/4.0/).

including their type of target. For example, Cas9 and Cas12 target DNA, whereas Cas13 targets RNA. While DNA-targeting enzymes are widely used for gene editing, RNA-targeting systems, like Cas13, are particularly valuable for modulating gene expression and combating RNA viruses.[31,32] Cas13d endonuclease is guided by a CRISPR-associated RNA (crRNA) to an RNA target in a sequence-specific manner. Upon target recognition, Cas13 cleaves the RNA, leading to its degradation. Additionally, Cas13 exhibits collateral activity *in vitro* and in bacteria, cleaving nearby non-target RNAs, which can be harnessed for applications such as viral detection.[33] This work focuses on employing the RNA-targeting CRISPR-Cas system, Cas13, as an antiviral as well as diagnostic method for human coronavirus infections. Recent studies have demonstrated that the LbuCas13a endonuclease (1,160 amino acids, aa) can inhibit the replication of influenza and SARS-CoV-2 by targeting the viral RNA genome. Similarly, the PspCas13b endonuclease (1,133 aa) has proven effective in suppressing SARS-CoV-2 replication.[34] Cas13d, a smaller variant of 967 aa, offers a significant size advantage over Cas13a-c.[35–37] This is particularly important for applications requiring delivery by viral vectors with a limited packaging capacity. Additionally, the Cas13d endonuclease demonstrates enhanced catalytic activity and higher cleavage efficiency compared to Cas13a and Cas13b, making it a potent tool for RNA-targeting applications.[38] Unlike other CRISPR systems, Cas13d does not rely on the constraint of a protospacer flanking sequence (PFS), allowing greater flexibility in target selection.[35,39] By targeting sequences that are highly conserved among different virus isolates, we aim not only to achieve broad-spectrum activity but also to minimize the risk of acquisition of viral escape mutations.[40,41] The ability of Cas13d to retain activity despite single mismatches in the crRNA-target duplex as long as they occur outside the crRNA seed region (distal positions 15–21 of the crRNA-target RNA duplex) makes it a powerful tool to combat rapidly mutating viral genomes, including coronaviruses, with potential applications in both antiviral treatment and diagnostic development.[42]

Our previous analysis of full-length viral genomes identified a highly conserved 26-nucleotides (nt) sequence (AUGGGUUGGGAUUAUC CUAAAUGUGA) within the nsp12 gene, encoding the viral RdRp, across all seven human coronaviruses (Figure 1A, red triangle).[43] In this study, we aim to assess the feasibility of using a single crRNA against this site to suppress the replication of all seven human coronaviruses. Additionally, we aim to repurpose this crRNA by developing a Cas13d-based diagnostic method. Rapid, accurate, and sensitive virus detection is crucial for effective monitoring of infectious diseases.[44] During the SARS-CoV-2 pandemic, laboratories worldwide developed a variety of platforms to detect this pathogen, with reverse-transcription PCR (RT-PCR) emerging as the gold standard for SARS-CoV-2 diagnostics.[45,46] However, this technique has notable limitations, including the need for expensive equipment and highly trained personnel. Additionally, the procedure often requires samples to be sent to centralized test facilities, such as local disease control centers. This process not only extends the turnaround time but also introduces additional risks, including contamination of the sample and spread of the pathogen. Therefore, accurate and simple point-of-care diagnostic testing is crucial for preventing and responding effectively to infectious disease outbreaks. *In vitro* nucleic acid-detection techniques based on CRISPR-Cas have been widely used.[47–50] The technique of “combining loop-mediated isothermal amplification” (LAMP) with CRISPR-Cas12a or recombinase polymerase amplification (RPA) with CRISPR-Cas13a and Cas12 has been employed to detect SARS-CoV-2.[51,52]

In this study, we aim to develop a broad-spectrum antiviral and diagnostic strategy based on Cas13d, which is well-suited for this purpose due to its lack of a PFS requirement. This feature expands the range of targetable RNA regions, including highly conserved regions that are less prone to changes due to evolutionary pressures, thereby supporting a robust diagnostic strategy.

RESULTS

Design of crRNAs against highly conserved coronavirus genome sequence

Cas13d demonstrated optimal cleavage efficiency with a crRNA of 23-nt.[42] We designed four overlapping crRNAs to target the 26-nt (AUGGGUUGGGAUUAUCCUAAAUGUGA) highly conserved sequence identified in our previous study (Figure 1B).[43] The mismatches for these crRNAs when annealed to the genomes of all seven human coronaviruses are highlighted in bold red (Figure 1C). The seed region of Cas13d/crRNA is indicated by a green box, spanning position 15–21, which is important for efficacy as mismatches in this region greatly affect the cleavage efficiency.[42] All crRNAs are fully complementary to the target sequences in SARS-CoV-2 and HCoV-HKU1, but a varying number of mismatches are present in the base-paired duplex with the RNA genome of other human coronaviruses (Figure 1C). For instance, the duplex formed by all four crRNAs with the HCoV-229E RNA genome contains three mismatches, with one or two located in the crRNA seed region. This seed region is critical for crRNA-target annealing and the initiation of cleavage, meaning that these mismatches could potentially reduce Cas13d endonuclease activity.[42] Additionally, the widely used Bowtie bioinformatics tool for aligning sequences with reference genomes was employed to identify potential off-target hits across the complete human transcriptome, including mature mRNA sequences. This *in silico* analysis indicated that the anti-coronaviral crRNAs we designed have no predicted off-target sites in the human transcriptome with three or fewer mismatches.

Cas13d-mediated targeting of the RNA genomes of all human coronaviruses

In order to assess the relative efficiency of different Cas13d/crRNAs in suppressing gene expression, the representative target sequences of crRNAs were cloned downstream of the luciferase open reading frame (Figure 2A). The luciferase reporter was co-transfected in human embryonic kidney (HEK) 293T cells with the expression vector encoding for both the crRNA and Cas13d endonuclease. The luciferase luminescence intensity was measured two days post-transfection. A non-targeting crRNA that does not recognize the human coronavirus genome, the expression vector, or the cell line genome

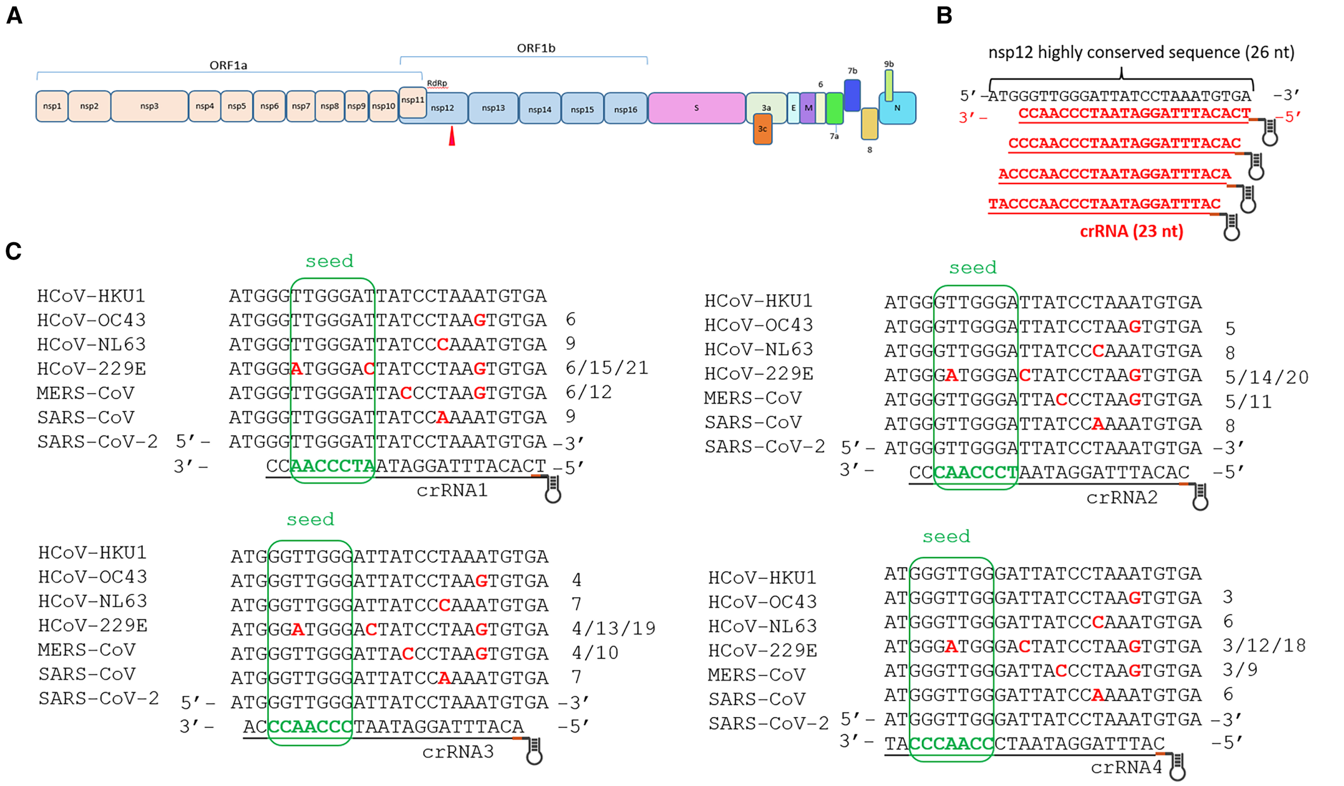

Figure 1. Schematic representation of the SARS-CoV-2 genome and crRNA design

(A) The SARS-CoV-2 genome is a single-stranded, positive-sense RNA. Its first two-thirds comprise overlapping open reading frames, ORF1a and ORF1b, which are translated into 16 nonstructural proteins (nsp1–nsp16). The remaining genome encodes structural proteins—spike (S), envelope (E), membrane (M), and nucleocapsid (N)—along with 8 accessory proteins: ORF3a, 3c, 6, 7a, 7b, 8, and 9b. (B) crRNA design. A highly conserved 26-nucleotide target region was identified within the viral genome of human coronaviruses. Based on this, four overlapping 23-nucleotides crRNAs were designed indicated in red. (C) The designed crRNAs were aligned to the genomes of all seven human coronavirus species. Mismatches relative to the SARS-CoV-2 reference sequence are highlighted in red. Each number on the right represents a single mismatch, with the value indicating its position within the target sequence. The Cas13d seed region, critical for target recognition and cleavage, is marked with a green rectangle.

was used as negative control, with its activity arbitrarily set at 100%. The crRNA targeting luciferase (Luc) was designed as positive control that targets luciferase RNA. The knockdown efficiency of all crRNAs across the seven human coronavirus reporters was assessed by measuring luciferase activity, as shown in Figure 2B. The numbers above the bars indicate the positions of mismatches, with the green numbers representing those located in the seed region. All crRNAs significantly inhibited Luciferase expression compared to the negative control ($p < 0.0001$). For the SARS-CoV-2 and HCoV-HKU1 reporter, where all crRNAs have a 100% sequence match, suppression of luciferase expression reached approximately 80%–90%. For SARS-CoV, HCoV-NL63, and HCoV-OC43, the four crRNAs achieved approximately 75%–80% luciferase suppression, indicating a tolerance of Cas13d for mismatches outside the seed region. Although a slight reduction in suppression was observed compared to SARS-CoV-2, it was not significant. Similarly, no significant differences were observed for the MERS-CoV reporter, which showed approximately 80% suppression, despite the presence of two mismatches in the crRNA-target duplex outside the seed region (Figure 1C). In contrast, for the HCoV-229E reporter with three mismatches, including one or two in the seed region, only a slight reduction in luciferase-suppression was measured for crRNA2 and crRNA3. These findings underscore the high tolerance of Cas13d for targets with 1–3 mismatches, particularly when mismatches are located outside the seed region. This suggests that even if the virus mutates to produce escape variants, these variants are unlikely to evade Cas13d, highlighting its strong potential for antiviral applications. A dose-dependent effect was observed, with increased crRNA levels showing more luciferase suppression (Figure S1). Although higher doses of crRNA were required to silence more divergent viruses compared to SARS-CoV-2, inhibition was still effectively achieved.

Efficient inhibition of human coronavirus replication by Cas13d with a single crRNA

We next aimed to evaluate the efficacy of these four crRNAs against the replicating virus, for which we used the replicon systems based on SARS-CoV-2, SARS-CoV, and MERS-CoV (Figure 3A). The SARS-CoV-2 infectious cDNA clone, based on the Wuhan Hu-1

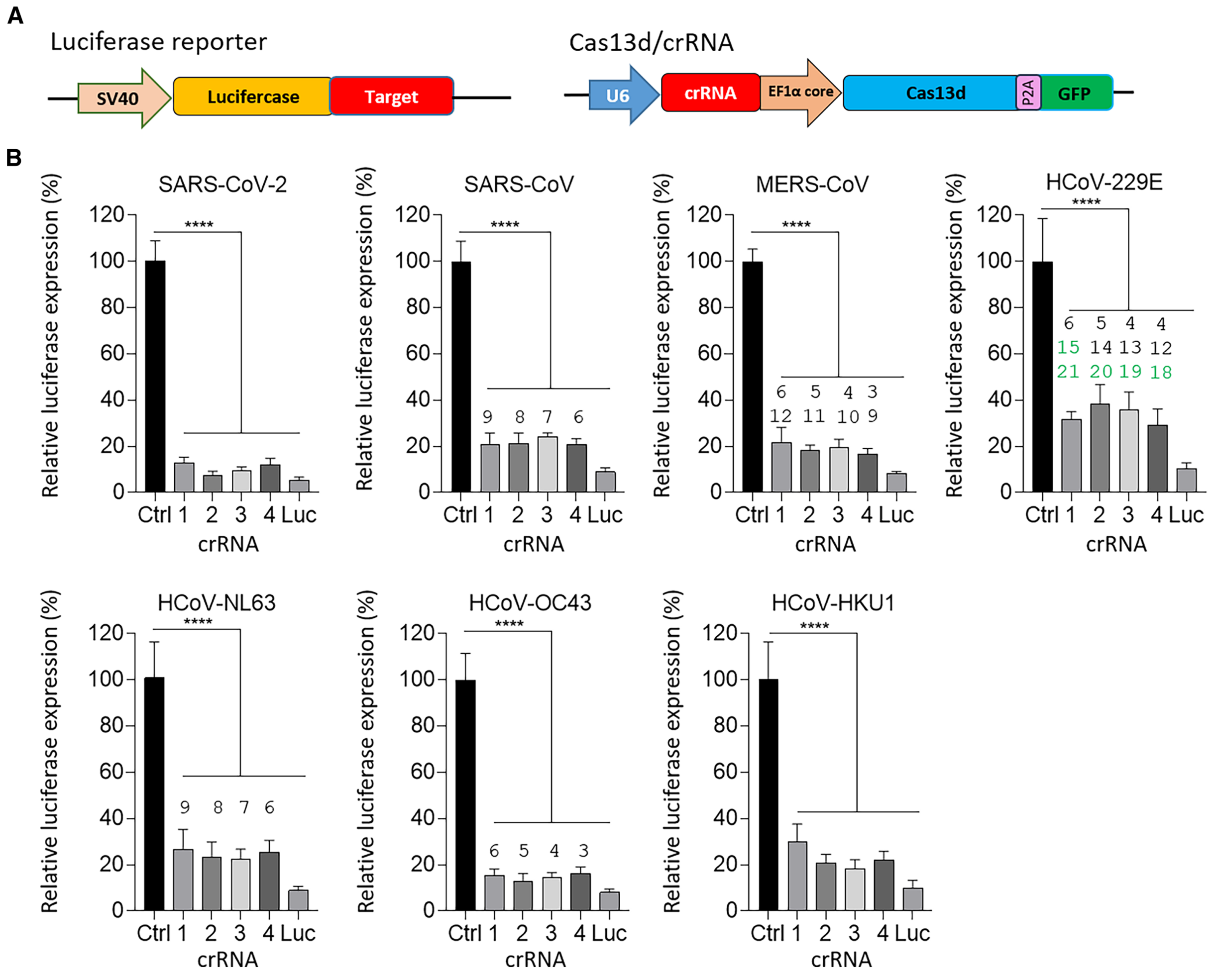

Figure 2. Broad antiviral CRISPR-Cas13d-crRNA activity against human coronaviruses genome

(A) Schematic diagrams illustrating the luciferase reporter system and the CRISPR-Cas13d expression cassette. The luciferase reporter gene is driven by the Pol II SV40 promoter, with the target sequence cloned downstream of the luciferase gene. The CRISPR-Cas13d expression cassette includes a U6 promoter for crRNA expression and an EF1α core promoter driving the expression of Cas13d and GFP. (B) Cas13d/crRNA construct was used to assess the inhibition of luciferase expression by various crRNAs. The tested crRNAs included those targeting the nsp12 genome (crRNA1-4) and a positive control Luc crRNA are shown relative to the Ctrl. Numbers above the bars indicate mismatch positions, with mismatches within the Cas13d seed region highlighted in green. Each number corresponds to a single mismatch. The data represent mean values (±SD) from three independent experiments each performed in duplicate. Statistical significance was analyzed using one-way ANOVA followed by Tukey's multiple comparisons test. Significant differences are indicated (****$p < 0.0001$).

strain (MN908947), was assembled in a bacterial artificial chromosome (BAC) as previously described.[53] To create the SARS-CoV-2 replicon (pBAC-SARS-CoV-2-mNG) that can be propagated without further security measures in BSL-2 laboratories, structural and genus-specific genes were replaced with the mNeonGreen (mNG) reporter gene, while the nucleocapsid (N) gene was retained for efficient RNA synthesis. A similar replicon was generated for the SARS-CoV Urbani strain (AY278741.1) and MERS-CoV EMC12 strain (JX869059.2).[53–55] Note that these replicons contain only the replicase gene, a reporter gene (if any), and N. These three replicons are capable of intracellular replication of the RNA genome but cannot produce infectious viral particles spreading to new cells. Following co-transfection of the viral replicons and Cas13d/crRNA constructs in HEK293T cells, the replication level was assessed by quantifying viral RNA synthesis using quantitative reverse-transcription PCR (RT-qPCR) at two days post-transfection. Total RNA was extracted from the cells and reverse transcribed into cDNA. The resulting cDNA was then used as a template for qPCR to measure the subgenomic mRNA N (sgmRNA-N) levels. The RNA level measured with the non-targeting negative control (Ctrl) was set at 100% in order to calculate the suppression measured for the four crRNAs (Figure 3B). All four crRNAs effectively inhibited replication of the SARS-CoV-2 replicon (>95% inhibition), demonstrating stronger suppression levels than those observed in the luciferase reporter system. We propose that the observed difference arises because luciferase readouts are buffered by translational

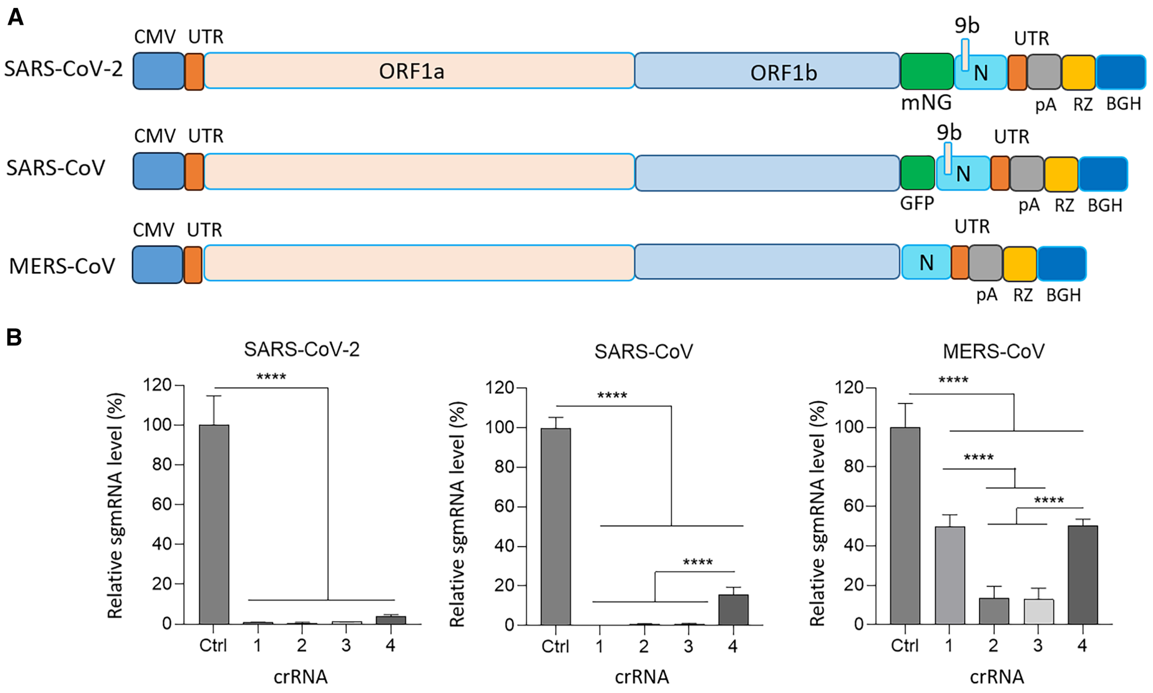

Figure 3. Cas13d broadly inhibits the replication of human coronavirus replicons

(A) Genomic diagrams of the replicons for SARS-CoV-2, SARS-CoV, and MERS-CoV. The replicons contain the 5′ and 3′ *cis*-acting signals required for viral replication, the large ORFs 1a and 1b encoding the replicase non-structural proteins (nsps), and the N gene, which is essential for efficient coronavirus RNA synthesis. The SARS-CoV-2 and SARS-CoV replicons, generated by *in vitro* ligation, also include a mNeonGreen (mNG) or GFP gene, respectively, positioned downstream of ORF1b and regulated by the S gene transcription regulatory sequence (TRS-M). (B) Inhibition of human coronavirus replicons by different crRNAs. Subgenomic mRNA (sgmRNA) levels were measured by RT-qPCR 2 days after transfection. A non-targeting crRNA (Ctrl) was included as a negative control. The horizontal axis shows the tested crRNAs, and the vertical axis shows relative viral RNA levels normalized to Ctrl. Data are presented as mean values (±SD) from three independent experiments each performed in duplicate. Statistical significance was determined using one-way ANOVA with Tukey's multiple comparisons test, with significance levels indicated as ****$p < 0.0001$.

amplification—allowing detectable protein even when Cas13d cleaves the RNA—whereas RT-qPCR directly measures sgmRNA, which is completely lost upon target cleavage, explaining the variation between protein-based and RNA-based measurements. For both the SARS-CoV and MERS-CoV replicons, mismatches in the crRNA-target duplex influenced inhibition efficiency. In the SARS-CoV replicon, a single mismatch occurs outside the seed region (Figure 1C), and all crRNAs reduced replication to levels comparable to SARS-CoV-2 (~95%), except for crRNA4, which showed slightly lower suppression (~80%). In the MERS-CoV replicon, two mismatches likely contributed to a more pronounced reduction in inhibitory activity, with inhibition ranging from ~50% to 80%. These observations suggest that factors, such as mismatch number, position, and local sequence context, can affect crRNA-target duplex stability, binding affinity, and target accessibility, although definitive conclusions require more systematic analysis.[34]

Next, we performed an infection experiment with the wild-type viruses HCoV-NL63 and HCoV-229E. To evaluate the impact of Cas13d/crRNA pre-treatment on viral replication, Huh-7 and LLC-MK2 cells were transduced with lentiviral constructs encoding the Cas13d endonuclease and individual crRNAs. Following transduction, the cells were sorted to ensure successful expression before proceeding to viral infection. Transduced Huh-7 and LLC-MK2 cells were infected with HCoV-229E and HCoV-NL63, respectively, at a multiplicity of infection (MOI) of 0.01. At two days post-infection (dpi), cells were collected, RNA was extracted, reverse transcribed, and quantified by qPCR. The non-targeting control (Ctrl) was set at 100%, and the sgmRNA-N levels in the presence of the four crRNAs were calculated relative to this control (Figure 4A). Despite the presence of three mismatches in the target sequence, all four crRNAs were capable of inhibiting HCoV-229E replication. Among them, crRNA1 was the least effective, with an inhibitory efficiency of approximately 40%, lower than the other crRNAs. This reduced efficacy is likely due to the presence of two mismatches within the seed region. In contrast, crRNA4 demonstrated the highest potency, achieving around 80% inhibition, while crRNA2 and crRNA3 showed intermediate efficiency of approximately 70%. For HCoV-NL63, which contains only a single mismatch outside the seed region, all crRNAs displayed strong antiviral activity with minor variation in their efficiencies. Overall, suppression levels against HCoV-NL63 were higher than those observed for HCoV-229E, with the exception of crRNA4, which showed a comparable level of inhibition in both viruses. Similar to the HCoV-229E results,

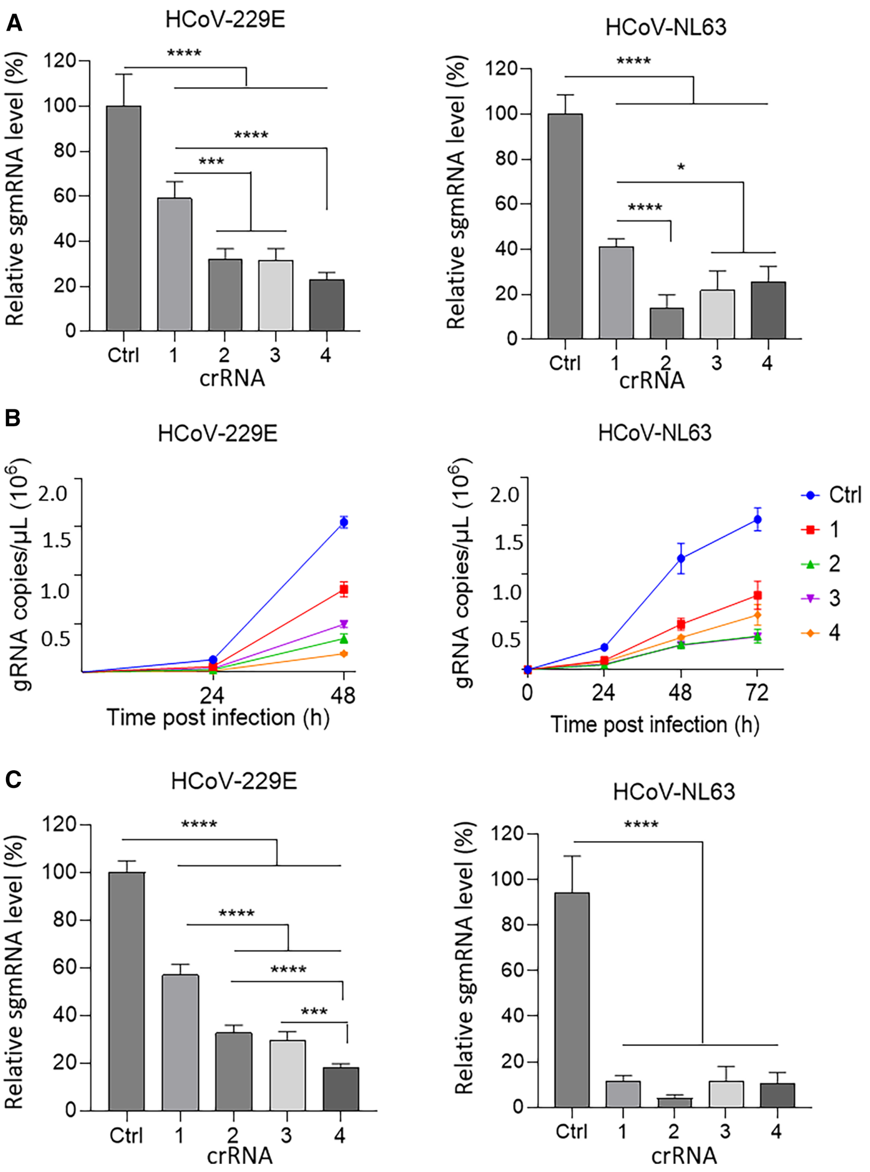

Figure 4. Cas13d inhibits the replication of various human coronavirus species when applied prior and post-infection

(A) Relative subgenomic mRNA (sgmRNA) levels at 2 days post-infection are shown as the ratio of viral RNA measured in the presence of different crRNAs relative to the control (Ctrl). (B) The antiviral effects of 4 crRNAs were evaluated by quantifying genomic RNA (gRNA) in the supernatant every 24 h, up to the peak of infection, with each crRNA represented by a different color. Viral gRNA levels, including those treated with the control crRNA, were quantified by RT-qPCR. (C) Relative sgmRNA levels are shown for cells treated with different Cas13d/crRNAs. A non-targeting crRNA (Ctrl) is included as a negative control and set to 100%. The horizontal axis indicates the tested crRNAs, and the vertical axis shows relative viral RNA expression. Data represent mean ± SD from three independent experiments. Statistical significance is indicated as *$p < 0.05$, **$p < 0.01$, ***$p < 0.001$, and ****$p < 0.0001$.

crRNA1 remained the least effective, with an inhibitory efficiency of around 60%, whereas crRNA2 was the most effective, reaching approximately 90% inhibition. Overall, inhibition was weaker against HCoV-229E than against HCoV-NL63, likely due to the higher number of mismatches between the crRNAs and their target sequences in the HCoV-229E genome. Viral genomic RNA levels in the culture supernatant for both HCoV-229E and HCoV-NL63 were then monitored over time (Figure 4B). For HCoV-229E, all tested crRNAs suppressed viral replication compared to the control, with inhibition levels of 87.6% (crRNA4), 77.6% (crRNA2), 68.1% (crRNA3), and 44.8% (crRNA1). For HCoV-NL63, the crRNAs also demonstrated inhibitory effects compared to the control, with inhibition levels of 78.0% (crRNA3), 77.6% (crRNA2), 63.6% (crRNA4), and 50.5% (crRNA1).

Alternatively, viral replication was initiated first at an MOI of 0.01, followed by subsequent treatment with Cas13d/crRNA to evaluate the therapeutic effect by measuring sgmRNA-N levels at 2 dpi (Figure 4C). All four crRNAs were able to inhibit viral replication in both HCoV-229E and HCoV-NL63 infections, with varying efficiencies. Against HCoV-229E, crRNA1 was the least effective, showing approximately 40% inhibition, whereas crRNA2, crRNA3, and crRNA4 achieved higher inhibition levels of approximately 70%–80%, with crRNA4 being the most potent. For HCoV-NL63, all crRNAs also demonstrated strong antiviral activity. crRNA2 showed the highest inhibition, exceeding 90%, while crRNA1, crRNA3, and crRNA4 maintained inhibition levels between 80% and 90%. These findings confirm that all tested crRNAs are capable of suppressing coronavirus replication and highlight the potential of the Cas13d system as a versatile and broad-spectrum antiviral strategy.

Cas13d/crRNA as a broad-spectrum diagnostic platform for human coronaviruses

Since these crRNAs target conserved viral sequences, they could potentially be adapted for use in CRISPR-based detection systems. Building on the specific high-sensitivity enzymatic reporter unlocking (SHERLOCK) technology, we re-engineered the workflow to use Cas13d instead of Cas13a, eliminating molecular constraints such as PFS in guide design. Cas13d retains target-activated collateral cleavage, enabling programmable detection of any RNA sequence of interest, including highly conserved sequences. Coupled with a lateral flow readout, our SHERLOCK-based assay allows for rapid, sensitive, and

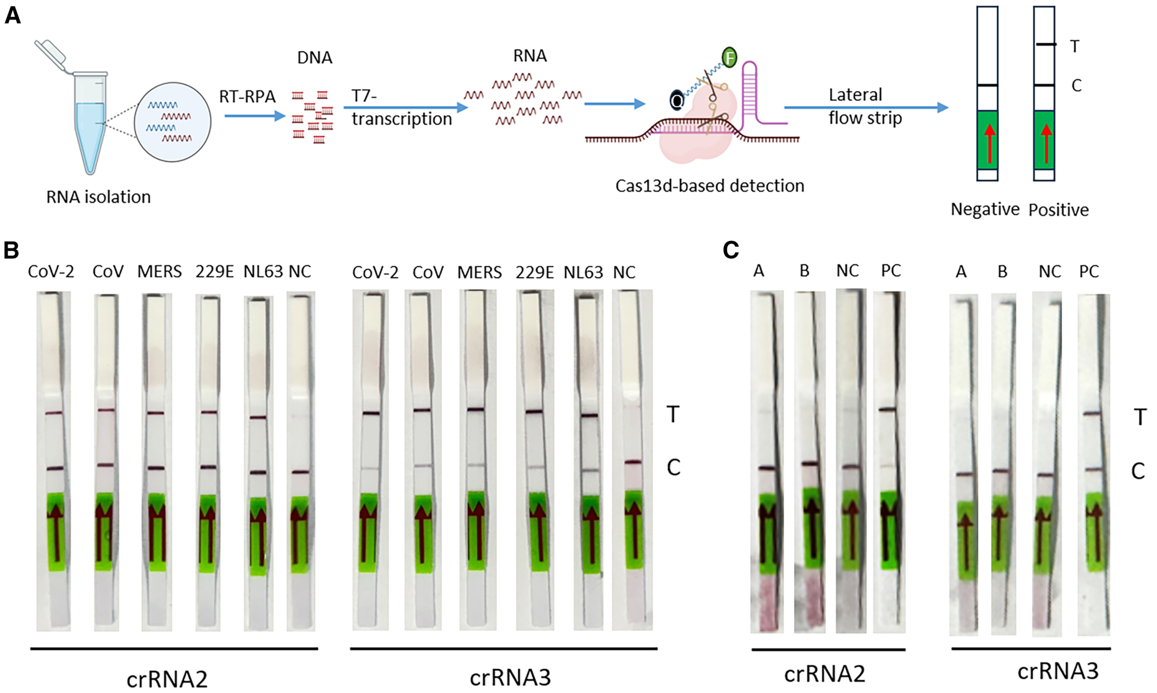

Figure 5. Cas13d-based SHERLOCK detection assay

(A) Detection workflow. Infectious samples are collected, and RNA is extracted. The extracted RNA is reverse transcribed into DNA and then amplified using RPA. The amplified DNA is subsequently transcribed by T7 RNA polymerase to generate RNA targets. These RNA targets are recognized and cleaved by CRISPR-Cas13d guided by crRNA. Upon activation, Cas13d triggers collateral cleavage activity, degrading all surrounding RNAs, including fluorescent RNA reporters. Detection is performed using a lateral flow strip, where a single band indicates a negative result and two bands indicate a positive result. (B) Broad detection of human coronaviruses using Cas13d. Either crRNA2 or crRNA3 was used to broadly detect five human coronavirus species: SARS-CoV-2, SARS-CoV, MERS-CoV, HCoV-229E, and HCoV-NL63. C, control line; T, test line; NC, negative control. (C) Specificity testing against non-coronavirus respiratory viruses. Influenza A and Influenza B samples were tested under the same conditions used for SARS-CoV-2 detection. C, control line; T, test line; PC, positive control (SARS-CoV-2).

design-flexible point-of-care diagnostics. First, we screened primers for reverse transcription-recombinase polymerase amplification (RT-RPA), an isothermal amplification technique that combines reverse transcription of RNA into cDNA with recombinase-mediated primer binding and DNA amplification. RPA operates at a constant temperature (37°C), eliminating the need for thermal cycling. A primer pair was identified (forward primer: 5′-CTAATACGACTCACTATAGGGATGGGTTGG-3′), reverse primer: 5′-ACACGTTGTATGTTTGCGAGCAAGAACAAG-3′) that amplifies the target region in the genomes of five human coronaviruses: SARS-CoV-2, SARS-CoV, MERS-CoV, HCoV-229E, and HCoV-NL63. A T7 RNA polymerase promoter sequence was incorporated at the 5′ end of the forward primer. The overall virus detection workflow is illustrated in Figure 5A. Briefly, RNA was extracted from HEK293T cells transfected with SARS-CoV-2, SARS-CoV, and MERS-CoV replicons, from Huh-7 cells infected with HCoV-229E and LLC-MK2 cells infected with HCoV-NL63. The extracted RNA was then subjected to reverse transcription and amplification using the selected RPA primers. The amplified products were then tested using the Cas13d-detection platform, which integrates *in vitro* transcription and Cas13d-based detection. Within this platform, we evaluated the performance of the two most effective crRNAs, crRNA2 and crRNA3.

The diagnostic results are presented as lateral flow strip outcomes in Figure 5B. A visible control line confirms the successful execution of the experiment, while the presence of a test line indicates a positive result. Both crRNA2 and crRNA3 consistently produced test lines for the detection of all five human coronaviruses, demonstrating their reliability as diagnostic tools. To further evaluate the specificity of our crRNAs and confirm that the SHERLOCK assay selectively detects coronaviruses, we tested their activity against other common respiratory viruses. Specifically, Influenza A and B strains were assessed under the same reaction conditions used for SARS-CoV-2 detection. No detectable signal was observed with these influenza viruses, whereas the SARS-CoV-2 positive control was consistently detected (Figure 5C). These results demonstrate the high specificity of our crRNAs for coronaviruses.

To quantify the analytical sensitivity of crRNA2 and crRNA3 across the five human coronaviruses, we tested a serial dilution of viral RNA copy numbers. Test-line intensity scaled with viral RNA copy number and the limit of detection (LoD) was defined as the lowest input yielding a signal above the negative-control threshold. As shown in Figure 6, crRNA2 and crRNA3 exhibited comparable sensitivity across all tested coronaviruses. Both crRNAs detected

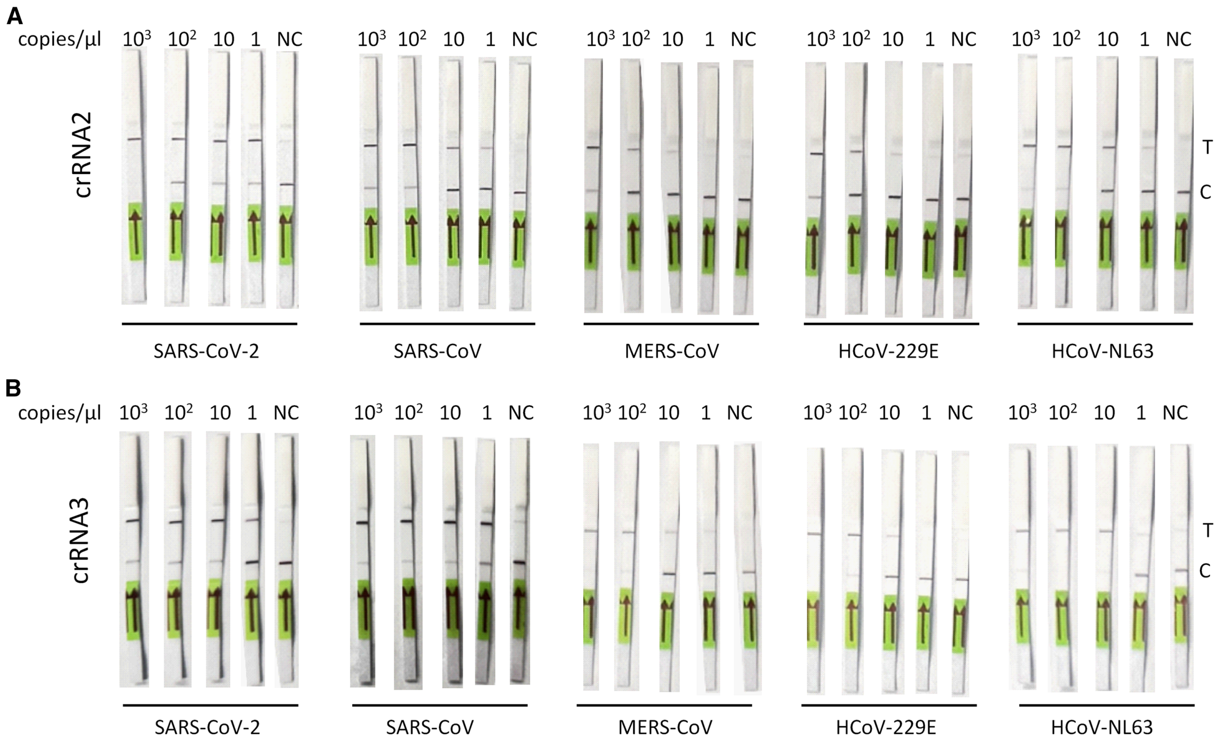

Figure 6. Limit of detection of crRNA2 and crRNA3 for various human coronaviruses
(A) Detection limit of crRNA2 and (B) crRNA3 for SARS-CoV-2, SARS-CoV, MERS-CoV, HCoV-229E, and HCoV-NL63. CRISPR-Cas13d, guided by a crRNA, specifically recognizes and cleaves the target RNA. Upon activation, Cas13d exhibits collateral cleavage activity, degrading nearby RNAs, including fluorescent reporters. Detection is carried out on a lateral flow strip: one band indicates a negative result, while two bands indicate a positive result. The assay was performed with serial dilutions (10-fold) ranging from 100 RNA copies down to 1 RNA copy. C, control line; T, test line; NC, negative control.

SARS-CoV-2 and SARS-CoV with a LoD of 1 copy/μL. For HCoV-NL63, the LoD was 10 copies/μL, while for MERS-CoV and HCoV-229E the LoDs were 100 copies/μL, consistent with the presence of three and two mismatches in their respective target sequences. These findings highlight crRNA2 and crRNA3 as promising candidates for the diagnosis of human coronavirus infection.

The conservation of the target sequence across animal coronavirus genomes

In the interest of pandemic preparedness, our research has focused on identifying a conserved sequence within human coronaviruses that can serve as a broad antiviral target and diagnostic tool for human coronaviruses. Recognizing the zoonotic origins of many viral outbreaks, we extended our *in silico* analysis to include coronaviruses in animal reservoirs. By aligning this highly conserved nsp12 sequence (AUGGGUUGGGAUUAUCCUAAAUGUGA) with corresponding sequences found across animal coronaviruses (Figures 7 and 8), we aim to develop a versatile tool that targets current human coronaviruses while offering potential for rapid adaptation in response to emerging zoonotic coronavirus threats. Four overlapping crRNAs, with all different seed regions, were designed, each 23 nt in length. The combined seed region of all four crRNAs is underlined in Figures 7 and 8. Nucleotides in the seed regions common to all crRNAs are highlighted in green. Figure 7 shows the mismatches between SARS-CoV-2 and various Bat-CoV isolates across the *Alphacoronavirus* and *Betacoronavirus* genera. The presence of highly conserved sequences highlights a shared genetic blueprint among these coronaviruses, enabling the targeting of regions common to multiple coronavirus strains. Some Bat-CoV sequences exhibit one or two mismatches outside the critical seed regions, suggesting that these isolates may still be effectively targeted by the same crRNAs designed for SARS-CoV-2. In contrast, sequences with one or two mismatches within the seed regions may require modification to maintain targeting specificity and efficacy. Notably, up to three mismatches, including one in the seed region, reduced both antiviral efficacy (Figure 4) and diagnostic sensitivity (Figures 5B and 6) but did not abolish either function. Figure 8 presents the mismatches between other animal coronaviruses and SARS-CoV-2. *Betacoronaviruses* show the highest conservation with SARS-CoV-2, with no mismatches in the crRNA seed region. *Gammacoronaviruses* display moderate similarity, with up to 3 mismatches, primarily outside the seed region. In contrast, *Alphacoronaviruses* and *Deltacoronaviruses* exhibit the least conservation, with mismatch counts reaching up to 5, mainly outside the seed region.

Genus	Specie	Sequence	Mismatches
Betacoronavirus	SARS-CoV-2	ATGGG**TTGG**GATTATCCTAAATGTGA	0
Alphacoronavirus and *Betacoronavirus*	Bat-CoV	ATGGGTTGGGATTATCCTAAATGTGA	0
		ATGGGTTGGGATTA**C**CCTAAATGTGA	1
		ATGGG**C**TGGGATTATCCTAAATGTGA	1
		ATGGGTTGGGATTATCC**A**AAATGTGA	1
		ATGGGTTGGGA**C**TATCCTAAATGTGA	1
		ATGGG**G**TGGGA**C**TATCCTAAATGTGA	2
		ATGGG**C**TGGGA**C**TATCCTAAATGTGA	2
		ATGGGTTGGGA**C**TA**C**CC**A**AAATGTGA	3
		ATGGGTTGGGATTA**C**CC**A**AAATGTGA	2
		ATGGGTTGGGATTATCC**A**AA**G**TGTGA	2
		ATGGGTTGGGATTATCCTAA**G**TGTGA	1
		ATGGG**A**TGGGATTATCCTAA**G**TGTGA	2
		ATGGGTTGGGA**C**TATCCTAA**G**TGTGA	2
		ATGGGTTGGGATTA**C**CCTAA**G**TGTGA	2
		ATGGG**A**TGGGATTA**C**CCTAA**G**TGTGA	3
		ATGGGTTGGGA**C**TA**C**CCTAA**G**TGTGA	3
		ATGGGTTGGGATTA**C**CCTAA**G**TG**C**GA	3

Figure 7. Mismatches of Bat-CoV relative to SARS-CoV-2 genome

Positions 15–21 of the crRNA correspond to the seed region (underlined in the SARS-CoV-2 reference sequence), which differs for each crRNA. Nucleotides conserved across all four crRNA seed regions are highlighted in green in the SARS-CoV-2 sequence, whereas mismatches present in Bat-CoV are shown in bold red. SARS-CoV-2, severe acute respiratory syndrome coronavirus 2; Bat-CoV, bat coronavirus.

These results suggest that SARS-CoV-2-targeted crRNAs may still be effective for antiviral applications and diagnostics across these animal coronaviruses but would likely require adjustments for antiviral applications. This still needs to be empirically tested.

DISCUSSION

We have previously identified a genomic region highly conserved across human coronaviruses within the nsp12 gene of ORF1ab.[43] The nsp12 protein, an RNA-dependent RNA polymerase, is essential for viral RNA synthesis and plays a central role in replication and transcription.[56,57] Due to its critical function, nsp12 serves as a primary target for various antiviral inhibitors, including remdesivir, which has shown significant therapeutic efficacy against SARS-CoV-2 infection.[58] In contrast, the CRISPR-Cas13d system presented here directly targets the viral RNA sequence, promoting degradation of the entire coronavirus gRNA rather than inhibiting a single viral protein, which could reduce the likelihood of resistance. Indeed, resistance to remdesivir has been observed as SARS-CoV-2 accumulates mutations in nsp12.[59] Ongoing viral transmission, continuous evolution, and selective pressures from drugs drive the emergence of resistant variants.[60–64] Additionally, coronaviruses comprise a diverse group with a broad host range, capable of infecting both humans and animals. These viruses can emerge through cross-species transmission from animals and subsequently adapt to spread among humans.[65] This evolutionary adaptability of coronaviruses challenges the effectiveness of current treatments and diagnostics, creating an urgent need for innovative strategies to detect and manage both existing and emerging strains.

CRISPR-Cas13d has proven to be an effective and robust antiviral system with high specificity.[35,66–69] Additionally, this system exhibits tolerance to mismatches, particularly when they occur outside the critical seed region.[42] In this study, we developed a Cas13d-based broad-spectrum effector targeting the highly conserved nsp12 sequence (AUGGGUUGGGAUUAUCCUAAAUGUGA) shared among human coronaviruses, as targeting conserved regions increases the barrier to viral escape. Our findings demonstrate that Cas13d crRNAs can achieve broad-spectrum inhibition of multiple human coronaviruses, with particularly high efficacy against SARS-CoV-2, SARS-CoV, HCoV-NL63, and HCoV-OC43. The robust inhibition observed, even in the presence of two mismatches for MERS-CoV targets, highlights the system's tolerance to sequence variations outside the seed region, suggesting potential utility for targeting diverse or rapidly evolving viral strains. In contrast, the reduced activity against HCoV-229E underscores the critical importance of seed-region complementarity for efficient target recognition and cleavage, consistent with previous mechanistic studies of Cas13 enzymes.[42,70,71] These observations suggest that careful crRNA design, prioritizing seed-region matches, is essential for maximizing antiviral activity, while also indicating that Cas13d may retain function against partially divergent viral sequences. Collectively, our results provide insights into the sequence specificity and mismatch tolerance of Cas13d, informing the rational design of broad-spectrum antiviral strategies and highlighting its potential as a versatile platform for combating emerging coronavirus threats.

Luciferase-based assays provide a rapid and effective method for selecting crRNAs and evaluating potential structural effects on activity. Although the reporter sequence is structurally simpler than the full viral genome, it does not reproduce the continuous RNA synthesis occurring during infection. To address this limitation and determine

Genus	Specie	Sequence	Mismatches
Betacoronavirus	SARS-CoV-2	ATGGGTTGGGATTATCCTAAATGTGA	0
Alphacoronavirus	PRCV	ATGGGATGGGACTATCCTAAGTGTGA	3
	CCoV	ATGGGATGGGACTATCCTAAGTGTGA	3
	PEDV	ATGGGTTGGGATTACCCAAAGTGCGA	4
		ATGGGTTGGGACTACCCAAAGTGCGA	5
	SADS-CoV	ATGGGCTGGGATTATCCTAAATGTGA	1
	FCoV	ATGGGGTGGGACTATCCTAAGTGTGA	3
		ATGGGATGGGACTATCCAAAGTGCGA	5
	FRCoV	ATGGGTTGGGATTACCCTAAGTGTGA	2
		ATGGGTTGGGATTACCCCAAGTGTGA	3
Betacoronavirus	MHV	ATGGGTTGGGACTATCCTAAATGTGA	1
		ATGGGTTGGGACTATCCTAAGTGTGA	2
	RCoV	ATGGGTTGGGATTATCCTAAATGTGA	0
		ATGGGTTGGGACTATCCTAAGTGTGA	2
	IBV	ATGGGTTGGGATTATCCAAAGTGTGA	2
		ATGGGTTGGGATTACCCAAAGTGTGA	3
		ATGGGTTGGGACTATCCAAAGTGTGA	3
	PHEV	ATGGGTTGGGATTATCCTAAATGTGA	0
		ATGGGTTGGGATTATCCAAAGTGTGA	2
	CRCoV	ATGGGTTGGGATTATCCTAAGTGTGA	1
	BCoV	ATGGGTTGGGATTATCCTAAGTGTGA	1
	ECoV	ATGGGTTGGGATTATCCTAAGTGTGA	1
		ATGGGTTGGGATTACCCTAAGTGTGA	2
Gammacoronavirus	TCoV	ATGGGTTGGGATTATCCTAAGTGTGA	1
		ATGGGTTGGGATTATCCAAAGTGTGA	2
	GCoV	ATGGGCTGGGACTATCCTAAATGTGA	2
		ATGGGCTGGGACTATCCAAAATGTGA	3
	DCoV	ATGGGTTGGGATTATCCTAAATGTGA	0
		ATGGGTTGGGATTATCCTAAGTGTGA	1
	BWCoV	ATGGGGTGGGACTATCCTAAGTGTGA	3
Deltacoronavirus	WCoV	TTTGGTTGGGATTATCCTAAATGTGA	2
	MHCoV	GCTGGTTGGGATTATCCTAAGTGTGA	4
	MCoV	GTGGGCTGGGATTACCCCAAGTGTGA	5
	THCoV	GTGGGTTGGGATTACCCTAAGTGTGA	3
	PDCoV	GTGGGTTGGGATTACCCTAAATGTGA	2
		GTGGGTTGGGATTACCCTAAGTGTGA	3

Figure 8. Mismatches of other animal coronaviruses relative to SARS-CoV-2 genome

Positions 15–21 of the crRNA correspond to the seed region (underlined in the SARS-CoV-2 reference sequence), which differs for each crRNA. Nucleotides conserved across all four crRNA seed regions are highlighted in green, whereas mismatches in the animal coronavirus sequences are shown in bold red. SARS-CoV-2, severe acute respiratory syndrome coronavirus 2; PRCV, porcine respiratory coronavirus; CCoV, canine coronavirus; PEDV, porcine epidemic diarrhea virus; SADS-CoV, swine acute diarrhea syndrome coronavirus; FCoV, feline coronavirus; FRCoV, ferret coronavirus; MHV, mouse hepatitis virus; RCoV, rat coronavirus; IBV, infectious bronchitis virus; PHEV, porcine hemagglutinating encephalomyelitis virus; CRCoV, canine respiratory coronavirus; BCoV, bovine coronavirus; ECoV, equine coronavirus; TCoV, turkey coronavirus; GCoV, goose coronavirus; DCoV, duck coronavirus; BWCoV, beluga whale coronavirus; WCoV, wigeon coronavirus; MHCoV, moorhen coronavirus; MCoV, munia coronavirus; THCoV, hrush coronavirus; PDCoV, porcine deltacoronavirus.

whether these crRNAs can achieve inhibition levels similar to those observed in the reporter system, we employed replicon systems for SARS-CoV-2, SARS-CoV, and MERS-CoV, along with replicating wild type viruses for HCoV-NL63 and HCoV-229E, to evaluate the antiviral efficacy of the selected crRNAs. For the SARS-CoV-2 replicon, the inhibition efficiency was slightly higher than that observed with the reporter system, with values exceeding 90% inhibition. Notably, crRNA4 showed similar inhibition of the SARS-CoV replicon compared to its performance in the reporter system, while the other three crRNAs even exceeded previous inhibition levels. For MERS-CoV, the inhibition efficiency of crRNA1 and crRNA4 in the replicon system decreased to approximately 50%, underscoring the importance of RNA accessibility and secondary structure in determining crRNA efficacy. In HCoV-NL63 replication experiments, the inhibitory efficiency of crRNA1 declined from ∼80% in the luciferase reporter assay to ∼60%. Similarly, in HCoV-229E

infection experiments, the efficiency of crRNA1 decreased from ~70% in the reporter assay to ~40%. This reduction in activity may be explained by the complex secondary structure of viral RNA or the presence of protective viral proteins, both of which could hinder crRNA binding and cleavage efficiency.[41] Therefore, it is essential to assess the effectiveness of crRNAs using replicable viral genomes.

We further evaluated the antiviral activity of Cas13d/crRNA against HCoV-229E and HCoV-NL63 virus, demonstrating that Cas13d programmed with a single crRNA significantly inhibited viral replication and retained therapeutic efficacy even after infection was established. Sequencing analysis after 2 weeks of viral passaging revealed no escape-associated mutations. This may be because mutations in these highly conserved sequences are not tolerated and would impose a growth disadvantage on the virus, or alternatively because the antiviral pressure was not sufficiently high to drive viral escape.

In summary, our study demonstrates the feasibility of targeting a highly conserved region across all seven human coronaviruses, enabling the use of a single crRNA for broad-spectrum inhibition. Unlike prior approaches that focused on strain-specific conserved sequences and required multiplexing multiple crRNAs for effective inhibition, our strategy simplifies therapeutic design while maintaining efficacy.[66] The exceptionally high conservation of our target sequence not only strengthens its potential as a robust antiviral target but also enhances future pandemic preparedness by providing a target likely to remain conserved in emerging coronaviruses, including those in animal reservoirs. While some adjustments may be needed for application in future pandemics, these modifications are straightforward and would allow rapid deployment against emerging viral threats.

Given Cas13d′s tolerance for mismatches, there is a potential risk of off-target activity within the human transcriptome.[71] To evaluate potential off-target effects, we performed an *in silico* Bowtie analysis, which revealed no sequences in the human transcriptome with three or fewer mismatches to the designed crRNAs, underscoring their predicted high specificity. Nevertheless, a thorough assessment of off-target activity will require experimental validation in future *in vivo* studies, such as transcriptome-wide profiling. In addition, Cas13d has been reported to exhibit collateral cleavage of non-target RNAs under certain conditions.[72] Although we did not detect measurable cytotoxicity or cell death in our experiments, assessing the relevance of collateral cleavage *in vivo* will be crucial to fully evaluate potential off-target RNA degradation and to establish the overall safety of Cas13d-based interventions. Recently developed high-fidelity Cas13 nucleases with reduced collateral activity may provide safer alternatives for therapeutic applications.[73]

On the other hand, the *in vitro* collateral activity is precisely what enables Cas13d to function in highly sensitive diagnostic applications. We developed a broad-spectrum diagnostic strategy using the same single crRNA to detect multiple human coronaviruses and validated its performance across five distinct strains, while showing no detectable activity against other respiratory viruses such as Influenza A and B. Furthermore, sensitivity assays showed that both crRNA2 and crRNA3 were capable of detecting as low as 1 cp/μL for SARS-CoV-2 and SARS-CoV, 10 cp/μL for HCoV-NL63, and 100 cp/μL for MERS-CoV and HCoV-229E. By targeting highly conserved regions of the viral genome, our assay ensures robust and sustained performance, minimizing the risk of obsolescence as the virus evolves. This approach also enables the detection of multiple coronaviruses using a single crRNA in a single assay, significantly reducing testing time, cost, and technical complexity. However, it should be noted that the current workflow still requires RNA extraction from biological samples, and direct detection from unprocessed specimens remains a technical challenge.

As we now know, SARS-CoV and MERS-CoV cause severe acute respiratory syndromes after crossing species barriers from natural animal reservoirs to humans.[12,74] Similarly, the other four human coronaviruses (HCoV-229E, HCoV-NL63, HCoV-OC43, and HCoV-HKU1) are also zoonotic in origin.[75,76] While the exact intermediate host of SARS-CoV-2 remains unknown, its close genomic similarity to bat coronavirus (Bat-CoV RaTG13), as well as to coronaviruses found in pangolins and raccoon dogs, suggests a potential zoonotic spillover from these species. Among them, current evidence points to raccoon dogs as the most likely intermediate host for SARS-CoV-2.[77–80] These recurring zoonotic transmissions highlight the importance of studying animal coronaviruses to better understand and predict future threats to human health.[81,82] To investigate this further, we analyzed the highly conserved region of the nsp12 gene in human coronaviruses and compared it with the corresponding regions in animal coronaviruses. Bats, known reservoirs for SARS-CoV and MERS-CoV,[83,84] are also likely reservoirs for SARS-CoV-2, with raccoon dogs serving as intermediate hosts.[85] Our analysis of Bat-CoV genomes revealed some genomes with perfect complementarity to SARS-CoV-2 in the highly conserved target region, while others showed up to three mismatches, with at most one mismatch located in the seed region in 5 out of 17 genome types. For other animal coronaviruses, the target region was highly conserved, with mutations consistently occurring at the same positions across different genome types. In some instances, identical mutations were observed in diverse genomes, highlighting the evolutionary conservation and functional significance of these regions. Our multifaceted analysis highlights the potential of CRISPR-Cas13d for diagnosing a wide range of animal coronaviruses, making it adaptable for both human and veterinary applications.

Overall, this study broadens the diagnostic and therapeutic toolbox for coronaviruses, providing a versatile and cost-effective potential countermeasure, for both current and emerging threats. Future studies focusing on *in vivo* validation and optimized delivery strategies will be essential to confirm efficacy and safety, and to enable rapid deployment against both current and emerging coronaviruses.

MATERIALS AND METHODS

crRNA design and plasmid construction

Four crRNAs were designed based on the previously identified 26-nt conserved sequence (AUGGGUUGGGAUUAUCCUAAAUGUGA), as shown in Figure 1B. Bowtie was used to identify crRNAs that align with the human transcriptome, allowing for up to three mismatches, to assess potential off-target effects.[86] The vector expressing RfxCas13d endonuclease (Addgene, #138147) was generously provided by Neville Sanjana (New York University, USA).[42] The plasmid was modified by replacing the puromycin resistance cassette with GFP to allow visualization. The U6 promoter was used to drive crRNA expression, while the EF1α core promoter was employed to express RfxCas13d, as depicted in Figure 2A. Forward and reverse oligonucleotides for each crRNA were annealed and ligated into the vector at the Esp3I restriction site. For the luciferase reporter, the target sequences from the seven human coronaviruses, along with 30 flanking bases on each side from each human coronavirus, were inserted into the pGL3-control vector using EcoRI and PstI restriction sites, as previously described.[87] The sequences inserted into the pGL3-control vector are listed in Table S1. The SV40 promoter was utilized to express luciferase, with the target sequences of each human coronavirus placed downstream of the luciferase expression cassette (Figure 2A).

The full-length cDNAs of the SARS-CoV-2 Wuhan Hu-1 strain (MN908947), the SARS-CoV Urbani strain, and MERS-CoV were cloned into bacterial artificial chromosomes (BAC), as previously reported.[53–55] To generate the replicons which can be safely used in BSL-2 laboratories without additional biosafety measures, the structural and genus-specific accessory genes were replaced with the GFP or mNG reporter gene or deleted, while the nucleocapsid (N) gene was retained to support efficient RNA synthesis (Figure 3A).

Cell culture and transfection

HEK293T cells were cultured in Dulbecco's modified Eagle's medium (DMEM) (Life Technologies, Invitrogen) supplemented with 10% fetal calf serum (FCS), 100 U/mL penicillin, 100 μg/mL streptomycin, and 1% L-glutamine. The cells were maintained in an incubator at 37°C with 5% CO_2. LLC-MK2 cells were cultured in minimum essential medium (MEM; Life Technologies, Invitrogen) supplemented with 10% FCS, 100 U/mL penicillin, 100 μg/mL streptomycin, and 1% non-essential amino acids (NEAA). Huh-7 cells were cultured in DMEM supplemented with 10% FCS, 100 U/mL penicillin, 100 μg/mL streptomycin, 1% NEAA, and 1% HEPES.

For luciferase assay experiments, HEK293T cells were seeded at a density of 1.4×10^5 cells/well in 24-well plates. Once cells reached approximately 70%–80% confluency, transfection was performed. For experiments testing different CRISPR-Cas13d/crRNAs, cells were transfected with 100 ng of the firefly luciferase reporter plasmid, 1 ng of renilla luciferase reporter plasmid (pRL), and 300 ng of CRISPR-Cas13d/crRNA. Two days post-transfection, CRISPR-Cas13d/crRNA editing efficiency was evaluated by measuring luciferase expression, as previously described.[87] In dose-dependent experiments, cells were transfected with 100 ng of luciferase expression plasmid, 1 ng of pRL, and 12, 60, or 300 ng of the CRISPR-Cas13d/crRNA2 construct. Each experiment was performed in triplicate, with two replicates per condition. Renilla luciferase activity was used to normalize transfection efficiency. A non-targeting crRNA (Ctrl) was used as the negative control, and its luciferase signal was set to 100%. The luciferase fluorescence signal intensity for each Cas13d/crRNA was calculated relative to the negative control. In addition, a crRNA specifically targeting luciferase (Luc) was included as a positive control.

RT-qPCR

Total RNA was extracted from collected cells or supernatants and reverse transcribed into cDNA using the High-Capacity cDNA Reverse Transcription Kit according to the manufacturer's protocol (Thermo Fisher Scientific). The resulting cDNA was stored at -20°C and used as the template for qPCR. To quantify viral subgenomic mRNA (sgmRNA) levels, the N gene sgmRNA was used because it is the most abundantly expressed viral mRNA and its production depends entirely on the activity of the viral RdRp, making it a reliable indicator of active viral replication and transcription. This is particularly relevant in our replicon system, where genomic RNA may also originate from the CMV promoter whereas sgmRNA-N synthesis requires functional RdRp activity. The 18S gene was used as an internal reference to normalize viral RNA levels against human cellular genomes. Viral RNA in the supernatant was also analyzed, as an indication of infectious viral load. The corresponding primers were used to amplify the cDNA of the five viruses, and the resulting PCR products were ligated into TA cloning vectors (Thermo Fisher Scientific) to generate standard templates for qPCR. Standard curves were prepared with a range of dilutions from 1.0×10^2 to 1.0×10^9 copies/μL across eight dilution points. The primers and probes used are listed in Table S2. qPCR was carried out using Platinum Quantitative PCR SuperMix-UDG (Invitrogen) and performed on a Rotor-Gene Q (QIAGEN) real-time system according to the manufacturer's instructions. The qPCR detection procedure was performed as previously described.[87]

Replicon assays

HEK293T cells were seeded into 24-well plates, and the following day, when confluency reached approximately 70%–80%, the cells were transfected with 300 ng of CRISPR-Cas13d/crRNA and 1.43 μg of replicon (SARS-CoV-2, SARS-CoV, or MERS-CoV) using Lipofectamine 2000 (Invitrogen) following the manufacturer's instructions, as previously described.[87] Three independent experiments were performed, each with two replicates. Two days post-transfection, the supernatant was discarded and the cells were collected. Total RNA was extracted from the cells and reverse transcribed into cDNA using the High-Capacity cDNA Reverse Transcription Kit (Thermo Fisher Scientific). The resulting cDNA was then used as the template for qPCR to measure the viral sgmRNA-N. A non-targeting crRNA (Ctrl) was used as the negative control, and the sgmRNA-N level was set to 100%.

Lentivirus production and transduction

The production and titration of lentiviral vectors were performed as described previously.[88] Briefly, lentiviral vector plasmids and packaging plasmids pSYNGP, pRSV-rev, and pVSV-g were transfected into HEK293T cells using Lipofectamine 2000 according to the manufacturer's instructions to produce lentivirus. Six hours post-transfection, the DMEM was replaced with OptiMEM (Invitrogen). Two days later, the supernatant was collected, centrifuged at 1,500 rpm for 5 min, and filtered through a 0.45-μm filter. The lentivirus was then concentrated 25-fold using Vivaspin 100,000 MWCO centrifugal filter units (Sartorius). To generate stable cell lines, LLC-MK2 or Huh-7 cells were transduced with serially diluted preparations of the concentrated lentivirus. Flow cytometry was performed 3 days post-transduction to quantify the proportion of GFP-positive cells. Cell populations with GFP-positive rate below 30% were selected for expansion to ensure 1 copy per cell. GFP-positive cells were sorted using a Sony SH800 sorter, expanded, and subsequently used for virus infection experiments.

Virus production and infection

LLC-MK2 and Huh-7 cells were seeded in T75 flasks. The following day, the media supplemented with 10% FCS was replaced with media containing 2% FCS, and the cells were inoculated with HCoV-NL63 or HCoV-229E, respectively. For viral propagation, the infected cells were incubated at 34°C with 5% CO_2. Once 80% cytopathic effect (CPE) was observed, the supernatant was collected, the cells were removed by low-speed centrifugation, and the viral supernatant was aliquoted and stored at −80°C for future use. The viral titer was determined using the 50% tissue culture infectious dose ($TCID_{50}$) assay, as previously described.[89] For infections, LLC-MK2 or Huh-7 cells were seeded at a density of 1×10^5 cells per well in 24-well plates. HCoV-NL63 or HCoV-229E were diluted in fresh media and inoculated onto the cells at an MOI of 0.01. The plates were incubated at 34°C, and cells were collected at 2 dpi for subsequent sgmRNA-N analysis. For the analysis of gRNA, viral supernatants were collected separately at 24, 48, and 72 h post-infection. Total RNA was subsequently extracted and used for qPCR analysis at the peak of infection. For viral escape evaluation, viral supernatants were collected at 72 h post-infection and continuously passaged twice per week. After 2 weeks of passaging, and upon completion of the final passage, the viral genome was sequenced to examine whether mutations had occurred in the targeted regions. Primer sequences are listed in Table S2.

Cas13d antiviral activity

To treat HCoV-NL63 or HCoV-229E infections, LLC-MK2 or Huh-7 cells were seeded into 24-well plates as previously described. After 24 h, the supernatant was removed, the cells were washed with PBS, and then infected at an MOI of 0.01. Three hours post-infection, the cells were washed with PBS, 1 mL fresh media was added, and the cells were transfected with 600 ng of the Cas13/crRNA complex. At 2 dpi, cells were collected, RNA was extracted and reverse transcribed, and the sgmRNA-N levels were quantified by qPCR.

Production of Cas13d protein and crRNA

E. coli Rosetta (*DE3*) was transformed with the Cas13d-expressing vector (Addgene, #171586) and cultured in Lysogeny Broth (LB) supplemented with kanamycin. The transformed cells were incubated in a shaker at 37°C for 12–16 h to establish a pre-culture. The pre-culture was transferred into 1 L of LB medium supplemented with kanamycin and incubated at 37°C with shaking. Optical density at 600 nm (OD600) was monitored every 15 min. When the OD600 reached 0.4–0.6, isopropyl-β-D-thiogalactopyranoside (IPTG) was added to a final concentration of 0.5 mM to induce protein expression. The culture was then incubated for an additional 4 h at 30°C with shaking.

Following incubation, the bacteria were harvested by centrifugation at 4,000 rpm for 20 min at 4°C, and the supernatant was discarded. The bacterial pellet was resuspended in lysis buffer (25 mM HEPES, pH 7.5; 600 mM NaCl; 5% glycerol; 10 mM imidazole; 1 mM TCEP) and homogenized until no visible cell clumps remained. The lysed cells were sonicated for 10 min at 40% amplitude while keeping the sample on ice. Following sonication, the lysate was centrifuged at 4,000 rpm for 10 min, and the supernatant was collected. The supernatant was then incubated with HisPur Ni-NTA Magnetic Beads (Thermo Fisher Scientific) on a roller at 4°C for 2 h to allow for His-tagged protein binding. The bead-protein mixture was transferred to Pierce Centrifuge Columns (Thermo Fisher Scientific r), washed with 50 mL of lysis buffer to remove non-specifically bound proteins, and the target protein was eluted with 5 mL of elution buffer (25 mM HEPES, pH 7.5; 600 mM NaCl; 5% glycerol; 100–300 mM imidazole; 1 mM TCEP). The purified protein was aliquoted and stored at −80°C to preserve stability for downstream applications. For crRNA production, the Invitrogen MAXIscript T7 Transcription Kit (Invitrogen) was used according to the manufacturer's instructions, with specific primers listed in Table S3.

SHERLOCK assays

RNA from the samples was extracted and reverse transcribed into cDNA, which served as the template for RPA-based isothermal amplification. The TwistAmp Basic Kit (TwistDx) was used for RPA, with modifications to the manufacturer's protocol. The master mix was prepared with the following reagents at final concentrations: 240 nM forward primer (5′-CTAATACGACTCACTATAGGGATGGGTTGG-3′), 240 nM reverse primer (5′-ACACGTTGTATGTTTGCGAGCAAGAACAAG-3′), 1× reaction buffer, and 100 μM betaine. The lyophilized reaction components from the TwistAmp Basic Kit were resuspended in the prepared master mix. cDNA templates were then added to the reaction, and 14 mM magnesium acetate ($C_4H_6MgO_4$) was added as the final component. The RPA reaction mixture was immediately incubated in a thermocycler at 37°C for 4 min, vortexed briefly, centrifuged, and then further incubated at 37°C for an additional 20 min. The resulting RPA products were subsequently used as substrates for detection. The fluorescent reporter was designed based on previous research.[90] For detection, the T7-FlashScribe Transcription Kit (Tebubio) was used to

transcribe RNA from the target region. The detection master mix included the following components: 8 mM HEPES (pH 6.8), 3.6 mM $MgCl_2$, 4 μM of each rNTP, 5.1 μg/mL Cas13d, 0.8 U/μL RNase inhibitor, 0.05 U/μL T7 RNA polymerase, 0.2 ng/μL crRNA, and 400 nM LF-RNA reporter. After adding the RPA products to the reaction, the mixture was vortexed, centrifuged, and incubated at 37°C in a PCR machine for 1 h.

For visualizing the results, the HybriDetect Universal Lateral Flow Assay Kit (Milenia Biotec) was used. A total of 20 μL of the detection solution was transferred into a 1.5-mL Eppendorf tube and mixed with 100 μL of HybriDetect buffer. The HybriDetect lateral flow strip was then placed in the Eppendorf tube. After incubating at room temperature for 3–5 min, the results were visible on the lateral flow strip.

Alignment of the genome sequences of animal coronaviruses

Complete genome sequences of coronaviruses from 23 animal species were obtained from the National Center for Biotechnology Information (NCBI) database. The SARS-CoV-2 Wuhan Hu-1 genome (GenBank: MN_908947) was used as the reference and aligned with each full-length animal coronavirus sequence using the multiple alignment program MAFFT. The aligned dataset was further analyzed in BioEdit to evaluate the sequence alignments with a specific sequence query.

Data analysis

Data were analyzed using GraphPad Prism (v.10.2.0; GraphPad Software, Inc., San Diego, CA, USA). For the transfection, replicon assay, and viral infection experiments, six replicates were used for each crRNA to calculate the standard deviation (SD), which is represented as error bars in the figures. Statistical analyses were performed using one-way ANOVA, followed by Tukey's multiple comparisons test to evaluate differences between groups. Significance levels were indicated as follows $*p < 0.05$; $**p < 0.01$; $***p < 0.001$; $****p < 0.0001$.

DATA AND CODE AVAILABILITY

All relevant data supporting the findings of this study are included in the article and supplemental materials or are available from the corresponding author upon request.

ACKNOWLEDGMENTS

Z.Y. and Y.B. are recipients of China Scholarship Council (CSC). E.H.-C. is recipient of Emergia Scientist Talent Attraction Program (DGP_EMEC_2023_00154). The HCoV-NL63 strains were obtained from Lia van der Hoek, and the replicons were provided by L.E. and S.Z. This work was supported by the Aspasia-NWO 015.015.040 grant. E.H.C.'s salary was partially covered by the Junta de Andalucía through the Emergia grant DGP_EMEC_2023_00154. We gratefully acknowledge Karen de Haan, Sarah van Leeuwen, and Colin Russell for kindly providing influenza samples used in the revision of this manuscript. The graphical abstract was created with BioRender.com.

AUTHOR CONTRIBUTIONS

E.H.-C. came up with the initial concept. Z.Y., E.H.-C., M.H., Y.B., and A.A.-L. designed the experiments. Z.Y., Y.B., A.A.L., and P.Z.K. conducted the experiments. Z.Z. conducted the Bowtie analysis. Z.Y., M.H., Y.B., A.A.L., and E.H.-C. analyzed the data. Z.Y., M.H., B.B., and E.H.-C. drafted the manuscript. All authors have read and agreed to the published version of the manuscript.

DECLARATION OF INTERESTS

The authors declare no conflict of interest.

SUPPLEMENTAL INFORMATION

Supplemental information can be found online at https://doi.org/10.1016/j.omtn.2026.102888.

REFERENCES

1. Fehr, A.R., and Perlman, S. (2015). Coronaviruses: An Overview of Their Replication and Pathogenesis. In Coronaviruses: Methods and Protocols, H.J. Maier, E. Bickerton, and P. Britton, eds. (Springer), pp. 1–23.
2. Woo, P.C.Y., Huang, Y., Lau, S.K.P., and Yuen, K.-Y. (2010). Coronavirus Genomics and Bioinformatics Analysis. Viruses *2*, 1804–1820.
3. Hamre, D., and Procknow, J.J. (1966). A New Virus Isolated from the Human Respiratory Tract. Proc. Soc. Exp. Biol. Med. *121*, 190–193.
4. McIntosh, K., Dees, J.H., Becker, W.B., Kapikian, A.Z., and Chanock, R.M. (1967). Recovery in tracheal organ cultures of novel viruses from patients with respiratory disease. Proc. Natl. Acad. Sci. USA *57*, 933–940.
5. van der Hoek, L., Pyrc, K., and Berkhout, B. (2006). Human coronavirus NL63, a new respiratory virus. FEMS Microbiol. Rev. *30*, 760–773.
6. van der Hoek, L., Pyrc, K., Jebbink, M.F., Vermeulen-Oost, W., Berkhout, R.J.M., Wolthers, K.C., Wertheim-van Dillen, P.M.E., Kaandorp, J., Spaargaren, J., and Berkhout, B. (2004). Identification of a new human coronavirus. Nat. Med. *10*, 368–373.
7. Walsh, E.E., Shin, J.H., and Falsey, A.R. (2013). Clinical impact of human coronaviruses 229E and OC43 infection in diverse adult populations. J. Infect. Dis. *208*, 1634–1642.
8. Garbino, J., Crespo, S., Aubert, J.D., Rochat, T., Ninet, B., Deffernez, C., Wunderli, W., Pache, J.C., Soccal, P.M., and Kaiser, L. (2006). A prospective hospital-based study of the clinical impact of non-severe acute respiratory syndrome (Non-SARS)-related human coronavirus infection. Clin. Infect. Dis. *43*, 1009–1015.
9. Vassilara, F., Spyridaki, A., Pothitos, G., Deliveliotou, A., and Papadopoulos, A. (2018). A Rare Case of Human Coronavirus 229E Associated with Acute Respiratory Distress Syndrome in a Healthy Adult. Case Rep. Infect. Dis. *2018*, 6796839.
10. van der Hoek, L., Sure, K., Ihorst, G., Stang, A., Pyrc, K., Jebbink, M.F., Petersen, G., Forster, J., Berkhout, B., and Uberla, K. (2005). Croup is associated with the novel coronavirus NL63. PLoS Med. *2*, e240.
11. Castillo, G., Mora-Díaz, J.C., Breuer, M., Singh, P., Nelli, R.K., and Giménez-Lirola, L.G. (2023). Molecular mechanisms of human coronavirus NL63 infection and replication. Virus Res. *327*, 199078.
12. Haagmans, B.L., Al Dhahiry, S.H.S., Reusken, C.B.E.M., Raj, V.S., Galiano, M., Myers, R., Godeke, G.J., Jonges, M., Farag, E., Diab, A., et al. (2014). Middle East respiratory syndrome coronavirus in dromedary camels: an outbreak investigation. Lancet Infect. Dis. *14*, 140–145.
13. Guan, Y., Zheng, B.J., He, Y.Q., Liu, X.L., Zhuang, Z.X., Cheung, C.L., Luo, S.W., Li, P.H., Zhang, L.J., Guan, Y.J., et al. (2003). Isolation and Characterization of Viruses Related to the SARS Coronavirus from Animals in Southern China. Science *302*, 276–278.
14. Wu, F., Zhao, S., Yu, B., Chen, Y.-M., Wang, W., Song, Z.-G., Hu, Y., Tao, Z.-W., Tian, J.-H., Pei, Y.-Y., et al. (2020). A new coronavirus associated with human respiratory disease in China. Nature *579*, 265–269.
15. Spinelli, A., and Pellino, G. (2020). COVID-19 pandemic: perspectives on an unfolding crisis. Br. J. Surg. *107*, 785–787.
16. Ahn, D.G., Shin, H.J., Kim, M.H., Lee, S., Kim, H.S., Myoung, J., Kim, B.T., and Kim, S.J. (2020). Current Status of Epidemiology, Diagnosis, Therapeutics, and Vaccines for Novel Coronavirus Disease 2019 (COVID-19). J. Microbiol. Biotechnol. *30*, 313–324.
17. Chen, Y., Liu, Q., and Guo, D. (2020). Emerging coronaviruses: Genome structure, replication, and pathogenesis. J. Med. Virol. *92*, 418–423.

18. Chen, S., Zhang, L., Wang, L., Ouyang, H., and Ren, L. (2021). Viruses from poultry and livestock pose continuous threats to human beings. Proc. Natl. Acad. Sci. USA *118*, e2022344118.

19. Forni, D., Cagliani, R., Clerici, M., and Sironi, M. (2017). Molecular Evolution of Human Coronavirus Genomes. Trends Microbiol. *25*, 35–48.

20. Pagani, I., Ghezzi, S., Alberti, S., Poli, G., and Vicenzi, E. (2023). Origin and evolution of SARS-CoV-2. Eur. Phys. J. Plus *138*, 157.

21. Damas, J., Hughes, G.M., Keough, K.C., Painter, C.A., Persky, N.S., Corbo, M., Hiller, M., Koepfli, K.P., Pfenning, A.R., Zhao, H., et al. (2020). Broad Host Range of SARS-CoV-2 Predicted by Comparative and Structural Analysis of ACE2 in Vertebrates. Preprint at bioRxiv. https://doi.org/10.1101/2020.04.16.045302.

22. Zhou, Z., Qiu, Y., and Ge, X. (2021). The taxonomy, host range and pathogenicity of coronaviruses and other viruses in the Nidovirales order. Anim. Dis. *1*, 5.

23. Zhou, P., and Shi, Z.L. (2021). SARS-CoV-2 spillover events. Science *371*, 120–122.

24. Koff, W.C., and Berkley, S.F. (2021). A universal coronavirus vaccine. Science *371*, 759.

25. Giurgea, L.T., Han, A., and Memoli, M.J. (2020). Universal coronavirus vaccines: the time to start is now. npj Vaccines *5*, 43.

26. Kostyushev, D., Kostyusheva, A., Ponomareva, N., Brezgin, S., and Chulanov, V. (2022). CRISPR/Cas and Hepatitis B Therapy: Technological Advances and Practical Barriers. Nucleic Acid Ther. *32*, 14–28.

27. Jena, R., Vishwas, S., Kumar, R., Kaur, J., Khursheed, R., Gulati, M., Singh, T.G., Vanathi, B.M., Alam, A., Kumar, B., et al. (2022). Treatment strategies for HIV infection with emphasis on role of CRISPR/Cas9 gene: Success so far and road ahead. Eur. J. Pharmacol. *931*, 175173.

28. Freen-van Heeren, J.J. (2022). Closing the Door with CRISPR: Genome Editing of CCR5 and CXCR4 as a Potential Curative Solution for HIV. BioTech. *11*, 25.

29. Li, P.Y., Li, S.Q., Gao, S.G., and Dong, D.Y. (2022). A one-step platform for screening high-efficient and minimal off-target CRISPR/Cas13 crRNAs to eradicate SARS-CoV-2 virus for treatment of COVID-19 patients. Med. Hypotheses *159*, 110754.

30. Hsu, P.D., Lander, E.S., and Zhang, F. (2014). Development and applications of CRISPR-Cas9 for genome engineering. Cell *157*, 1262–1278.

31. Freije, C.A., Myhrvold, C., Boehm, C.K., Lin, A.E., Welch, N.L., Carter, A., Metsky, H.C., Luo, C.Y., Abudayyeh, O.O., Gootenberg, J.S., et al. (2019). Programmable Inhibition and Detection of RNA Viruses Using Cas13. Mol. Cell *76*, 826–837.e11.

32. Blanchard, E.L., Vanover, D., Bawage, S.S., Tiwari, P.M., Rotolo, L., Beyersdorf, J., Peck, H.E., Bruno, N.C., Hincapie, R., Michel, F., et al. (2021). Treatment of influenza and SARS-CoV-2 infections via mRNA-encoded Cas13a in rodents. Nat. Biotechnol. *39*, 717–726.

33. Ackerman, C.M., Myhrvold, C., Thakku, S.G., Freije, C.A., Metsky, H.C., Yang, D.K., Ye, S.H., Boehm, C.K., Kosoko-Thoroddsen, T.S.F., Kehe, J., et al. (2020). Massively multiplexed nucleic acid detection with Cas13. Nature *582*, 277–282.

34. Fareh, M., Zhao, W., Hu, W., Casan, J.M.L., Kumar, A., Symons, J., Zerbato, J.M., Fong, D., Voskoboinik, I., Ekert, P.G., et al. (2021). Reprogrammed CRISPR-Cas13b suppresses SARS-CoV-2 replication and circumvents its mutational escape through mismatch tolerance. Nat. Commun. *12*, 4270.

35. Konermann, S., Lotfy, P., Brideau, N.J., Oki, J., Shokhirev, M.N., and Hsu, P.D. (2018). Transcriptome Engineering with RNA-Targeting Type VI-D CRISPR Effectors. Cell *173*, 665–676.e14.

36. Yan, W.X., Chong, S., Zhang, H., Makarova, K.S., Koonin, E.V., Cheng, D.R., and Scott, D.A. (2018). Cas13d Is a Compact RNA-Targeting Type VI CRISPR Effector Positively Modulated by a WYL-Domain-Containing Accessory Protein. Mol. Cell *70*, 327–339.e5.

37. Kushawah, G., Hernandez-Huertas, L., Abugattas-Nuñez Del Prado, J., Martinez-Morales, J.R., DeVore, M.L., Hassan, H., Moreno-Sanchez, I., Tomas-Gallardo, L., Diaz-Moscoso, A., Monges, D.E., et al. (2020). CRISPR-Cas13d Induces Efficient mRNA Knockdown in Animal Embryos. Dev. Cell *54*, 805–817.e807.

38. Zhang, C., Konermann, S., Brideau, N.J., Lotfy, P., Wu, X., Novick, S.J., Strutzenberg, T., Griffin, P.R., Hsu, P.D., and Lyumkis, D. (2018). Structural Basis for the RNA-Guided Ribonuclease Activity of CRISPR-Cas13d. Cell *175*, 212–223.e17.

39. Cox, D.B.T., Gootenberg, J.S., Abudayyeh, O.O., Franklin, B., Kellner, M.J., Joung, J., and Zhang, F. (2017). RNA editing with CRISPR-Cas13. Science *358*, 1019–1027.

40. Lin, X., Liu, Y., Chemparathy, A., Pande, T., La Russa, M., and Qi, L.S. (2021). A comprehensive analysis and resource to use CRISPR-Cas13 for broad-spectrum targeting of RNA viruses. Cell Rep. Med. *2*, 100245.

41. Abbott, T.R., Dhamdhere, G., Liu, Y., Lin, X., Goudy, L., Zeng, L., Chemparathy, A., Chmura, S., Heaton, N.S., Debs, R., et al. (2020). Development of CRISPR as an Antiviral Strategy to Combat SARS-CoV-2 and Influenza. Cell *181*, 865–876.e12.

42. Wessels, H.H., Méndez-Mancilla, A., Guo, X., Legut, M., Daniloski, Z., and Sanjana, N.E. (2020). Massively parallel Cas13 screens reveal principles for guide RNA design. Nat. Biotechnol. *38*, 722–727.

43. Hussein, M., Andrade Dos Ramos, Z., Berkhout, B., and Herrera-Carrillo, E. (2022). In Silico Prediction and Selection of Target Sequences in the SARS-CoV-2 RNA Genome for an Antiviral Attack. Viruses *14*, 385.

44. Zhou, P., Yang, X.L., Wang, X.G., Hu, B., Zhang, L., Zhang, W., Si, H.R., Zhu, Y., Li, B., Huang, C.L., et al. (2020). A pneumonia outbreak associated with a new coronavirus of probable bat origin. Nature *579*, 270–273.

45. Safiabadi Tali, S.H., LeBlanc, J.J., Sadiq, Z., Oyewunmi, O.D., Camargo, C., Nikpour, B., Armanfard, N., Sagan, S.M., and Jahanshahi-Anbuhi, S. (2021). Tools and Techniques for Severe Acute Respiratory Syndrome Coronavirus 2 (SARS-CoV-2)/COVID-19 Detection. Clin. Microbiol. Rev. *34*, e00228-20.

46. LeBlanc, J.J., Gubbay, J.B., Li, Y., Needle, R., Arneson, S.R., Marcino, D., Charest, H., Desnoyers, G., Dust, K., Fattouh, R., et al. (2020). Real-time PCR-based SARS-CoV-2 detection in Canadian laboratories. J. Clin. Virol. *128*, 104433.

47. Li, L., Li, S., Wu, N., Wu, J., Wang, G., Zhao, G., and Wang, J. (2019). HOLMESv2: A CRISPR-Cas12b-Assisted Platform for Nucleic Acid Detection and DNA Methylation Quantitation. ACS Synth. Biol. *8*, 2228–2237.

48. Chen, J.S., Ma, E., Harrington, L.B., Da Costa, M., Tian, X., Palefsky, J.M., and Doudna, J.A. (2018). CRISPR-Cas12a target binding unleashes indiscriminate single-stranded DNase activity. Science *360*, 436–439.

49. Gootenberg, J.S., Abudayyeh, O.O., Kellner, M.J., Joung, J., Collins, J.J., and Zhang, F. (2018). Multiplexed and portable nucleic acid detection platform with Cas13, Cas12a, and Csm6. Science *360*, 439–444.

50. Gootenberg, J.S., Abudayyeh, O.O., Lee, J.W., Essletzbichler, P., Dy, A.J., Joung, J., Verdine, V., Donghia, N., Daringer, N.M., Freije, C.A., et al. (2017). Nucleic acid detection with CRISPR-Cas13a/C2c2. Science *356*, 438–442.

51. Sun, Y., Yu, L., Liu, C., Ye, S., Chen, W., Li, D., and Huang, W. (2021). One-tube SARS-CoV-2 detection platform based on RT-RPA and CRISPR/Cas12a. J. Transl. Med. *19*, 74.

52. Arizti-Sanz, J., Freije, C.A., Stanton, A.C., Petros, B.A., Boehm, C.K., Siddiqui, S., Shaw, B.M., Adams, G., Kosoko-Thoroddsen, T.S.F., Kemball, M.E., et al. (2020). Streamlined inactivation, amplification, and Cas13-based detection of SARS-CoV-2. Nat. Commun. *11*, 5921.

53. Wang, L., Guzman, M., Muñoz-Santos, D., Honrubia, J.M., Ripoll-Gomez, J., Delgado, R., Sola, I., Enjuanes, L., and Zuñiga, S. (2023). Cell type dependent stability and virulence of a recombinant SARS-CoV-2, and engineering of a propagation deficient RNA replicon to analyze virus RNA synthesis. Front. Cell. Infect. Microbiol. *13*, 1268227.

54. Almazán, F., DeDiego, M.L., Sola, I., Zuñiga, S., Nieto-Torres, J.L., Marquez-Jurado, S., Andrés, G., and Enjuanes, L. (2013). Engineering a replication-competent, propagation-defective Middle East respiratory syndrome coronavirus as a vaccine candidate. mBio *4*, e00650.

55. Almazán, F., Dediego, M.L., Galán, C., Escors, D., Alvarez, E., Ortego, J., Sola, I., Zuñiga, S., Alonso, S., Moreno, J.L., et al. (2006). Construction of a severe acute respiratory syndrome coronavirus infectious cDNA clone and a replicon to study coronavirus RNA synthesis. J. Virol. *80*, 10900–10906.

56. Subissi, L., Posthuma, C.C., Collet, A., Zevenhoven-Dobbe, J.C., Gorbalenya, A.E., Decroly, E., Snijder, E.J., Canard, B., and Imbert, I. (2014). One severe acute respiratory syndrome coronavirus protein complex integrates processive RNA polymerase and exonuclease activities. Proc. Natl. Acad. Sci. USA *111*, E3900–E3909.

57. Yang, L., Zeng, X.T., Luo, R.H., Ren, S.X., Liang, L.L., Huang, Q.X., Tang, Y., Fan, H., Ren, H.Y., Zhang, W.J., et al. (2024). SARS-CoV-2 NSP12 utilizes various host splicing factors for replication and splicing regulation. J. Med. Virol. *96*, e29396.

58. Wang, M., Cao, R., Zhang, L., Yang, X., Liu, J., Xu, M., Shi, Z., Hu, Z., Zhong, W., and Xiao, G. (2020). Remdesivir and chloroquine effectively inhibit the recently emerged novel coronavirus (2019-nCoV) in vitro. Cell Res. *30*, 269–271.

59. Stevens, L.J., Pruijssers, A.J., Lee, H.W., Gordon, C.J., Tchesnokov, E.P., Gribble, J., George, A.S., Hughes, T.M., Lu, X., Li, J., et al. (2022). Mutations in the SARS-CoV-2 RNA-dependent RNA polymerase confer resistance to remdesivir by distinct mechanisms. Sci. Transl. Med. *14*, eabo0718.

60. Moghadasi, S.A., Heilmann, E., Khalil, A.M., Nnabuife, C., Kearns, F.L., Ye, C., Moraes, S.N., Costacurta, F., Esler, M.A., Aihara, H., et al. (2023). Transmissible SARS-CoV-2 variants with resistance to clinical protease inhibitors. Sci. Adv. *9*, eade8778.

61. Beeraka, N.M., Sukocheva, O.A., Lukina, E., Liu, J., and Fan, R. (2022). Development of antibody resistance in emerging mutant strains of SARS CoV-2: Impediment for COVID-19 vaccines. Rev. Med. Virol. *32*, e2346.

62. Guo, H., Fan, Q., Song, S., Shen, S., Zhou, B., Wang, H., Cheng, L., Ge, X., Ju, B., and Zhang, Z. (2022). Increased resistance of SARS-CoV-2 Lambda variant to antibody neutralization. J. Clin. Virol. *150–151*, 105162.

63. Chakraborty, C., Bhattacharya, M., and Sharma, A.R. (2022). Emerging mutations in the SARS-CoV-2 variants and their role in antibody escape to small molecule-based therapeutic resistance. Curr. Opin. Pharmacol. *62*, 64–73.

64. Hoffman, S.A., Costales, C., Sahoo, M.K., Palanisamy, S., Yamamoto, F., Huang, C., Verghese, M., Solis, D.A., Sibai, M., Subramanian, A., et al. (2021). SARS-CoV-2 Neutralization Resistance Mutations in Patient with HIV/AIDS, California, USA. Emerg. Infect. Dis. *27*, 2720–2723.

65. Liu, Y.C., Kuo, R.L., and Shih, S.R. (2020). COVID-19: The first documented coronavirus pandemic in history. Biomed. J. *43*, 328–333.

66. Zeng, L., Liu, Y., Wrynla, X.H., Abbott, T.R., Han, M., Zhu, Y., Chemparathy, A., Lin, X., Chen, X., Wang, H., et al. (2022). Broad-spectrum CRISPR-mediated inhibition of SARS-CoV-2 variants and endemic coronaviruses in vitro. Nat. Commun. *13*, 2766.

67. Liu, Z., Gao, X., Kan, C., Li, L., Zhang, Y., Gao, Y., Zhang, S., Zhou, L., Zhao, H., Li, M., et al. (2023). CRISPR-Cas13d effectively targets SARS-CoV-2 variants, including Delta and Omicron, and inhibits viral infection. MedComm *4*, e208.

68. Kuo, H.C., Prupes, J., Chou, C.W., and Finkelstein, I.J. (2024). Massively parallel profiling of RNA-targeting CRISPR-Cas13d. Nat. Commun. *15*, 498.

69. De Carli, A., Favaro, D., Filipponi, C., Filippini, F., Fonnesu, R., Plicanti, E., Nottoli, S., Barski, P., Lindstaedt, A., Witt, D., et al. (2025). Fighting RNA viruses with a gold nanoparticle Cas13d gene-editing armor. Mol. Ther. Nucleic Acids *36*, 102540.

70. Mayes, C.M., and Santarpia, J.L. (2023). Pan-Coronavirus CRISPR-CasRx Effector System Significantly Reduces Viable Titer in HCoV-OC43, HCoV-229E, and SARS-CoV-2. CRISPR J. *6*, 359–368.

71. Mayes, C.M., and Santarpia, J. (2022). Evaluating the Impact of gRNA SNPs in CasRx Activity for Reducing Viral RNA in HCoV-OC43. Cells *11*, 1859.

72. Shi, P., Murphy, M.R., Aparicio, A.O., Kesner, J.S., Fang, Z., Chen, Z., Trehan, A., Guo, Y., and Wu, X. (2023). Collateral activity of the CRISPR/RfxCas13d system in human cells. Commun. Biol. *6*, 334.

73. Tong, H., Huang, J., Xiao, Q., He, B., Dong, X., Liu, Y., Yang, X., Han, D., Wang, Z., Wang, X., et al. (2023). High-fidelity Cas13 variants for targeted RNA degradation with minimal collateral effects. Nat. Biotechnol. *41*, 108–119.

74. Shi, Z., and Hu, Z. (2008). A review of studies on animal reservoirs of the SARS coronavirus. Virus Res. *133*, 74–87.

75. Su, S., Wong, G., Shi, W., Liu, J., Lai, A.C.K., Zhou, J., Liu, W., Bi, Y., and Gao, G.F. (2016). Epidemiology, Genetic Recombination, and Pathogenesis of Coronaviruses. Trends Microbiol. *24*, 490–502.

76. Li, X., Song, Y., Wong, G., and Cui, J. (2020). Bat origin of a new human coronavirus: there and back again. Sci. China Life Sci. *63*, 461–462.

77. Poudel, U., Subedi, D., Pantha, S., and Dhakal, S. (2020). Animal coronaviruses and coronavirus disease 2019: Lesson for One Health approach. Open Vet. J. *10*, 239–251.

78. Worobey, M., Levy, J.I., Malpica Serrano, L., Crits-Christoph, A., Pekar, J.E., Goldstein, S.A., Rasmussen, A.L., Kraemer, M.U.G., Newman, C., Koopmans, M.P.G., et al. (2022). The Huanan Seafood Wholesale Market in Wuhan was the early epicenter of the COVID-19 pandemic. Science *377*, 951–959.

79. Pekar, J.E., Magee, A., Parker, E., Moshiri, N., Izhikevich, K., Havens, J.L., Gangavarapu, K., Malpica Serrano, L.M., Crits-Christoph, A., Matteson, N.L., et al. (2022). The molecular epidemiology of multiple zoonotic origins of SARS-CoV-2. Science *377*, 960–966.

80. Liu, W.J., Liu, P., Lei, W., Jia, Z., He, X., Shi, W., Tan, Y., Zou, S., Wong, G., Wang, J., et al. (2024). Surveillance of SARS-CoV-2 at the Huanan Seafood Market. Nature *631*, 402–408.

81. Lin, C.-N., Chan, K.R., Ooi, E.E., Chiou, M.-T., Hoang, M., Hsueh, P.-R., and Ooi, P.T. (2021). Animal Coronavirus Diseases: Parallels with COVID-19 in Humans. Viruses *13*, 1507.

82. Wang, N., Ji, W., Jiao, H., Veit, M., Sun, J., Wang, Y., Ma, X., Wang, Y., Wang, Y., Li, X.X., et al. (2025). A MERS-CoV-like mink coronavirus uses ACE2 as entry receptor. Nature *642*, 739–746.

83. Li, W., Shi, Z., Yu, M., Ren, W., Smith, C., Epstein, J.H., Wang, H., Crameri, G., Hu, Z., Zhang, H., et al. (2005). Bats are natural reservoirs of SARS-like coronaviruses. Science *310*, 676–679.

84. Dróżdż, M., Krzyżek, P., Dudek, B., Makuch, S., Janczura, A., and Paluch, E. (2021). Current State of Knowledge about Role of Pets in Zoonotic Transmission of SARS-CoV-2. Viruses *13*, 1149.

85. Lam, T.T.Y., Jia, N., Zhang, Y.W., Shum, M.H.H., Jiang, J.F., Zhu, H.C., Tong, Y.G., Shi, Y.X., Ni, X.B., Liao, Y.S., et al. (2020). Identifying SARS-CoV-2-related coronaviruses in Malayan pangolins. Nature *583*, 282–285.

86. Langmead, B., Trapnell, C., Pop, M., and Salzberg, S.L. (2009). Ultrafast and memory-efficient alignment of short DNA sequences to the human genome. Genome Biol. *10*, R25.

87. Hussein, M., Andrade Dos Ramos, Z., Vink, M.A., Kroon, P., Yu, Z., Enjuanes, L., Zuñiga, S., Berkhout, B., and Herrera-Carrillo, E. (2023). Efficient CRISPR-Cas13d-Based Antiviral Strategy to Combat SARS-CoV-2. Viruses *15*, 686.

88. ter Brake, O., Konstantinova, P., Ceylan, M., and Berkhout, B. (2006). Silencing of HIV-1 with RNA Interference: a Multiple shRNA Approach. Mol. Ther. *14*, 883–892.

89. Bullen, C.K., Davis, S.L., and Looney, M.M. (2022). Quantification of Infectious SARS-CoV-2 by the 50% Tissue Culture Infectious Dose Endpoint Dilution Assay. Methods Mol. Biol. *2452*, 131–146.

90. Kellner, M.J., Koob, J.G., Gootenberg, J.S., Abudayyeh, O.O., and Zhang, F. (2019). SHERLOCK: nucleic acid detection with CRISPR nucleases. Nat. Protoc. *14*, 2986–3012.

OMTN, Volume *37*

Supplemental information

Broad-spectrum CRISPR-Cas13d-mediated strategy for combating human coronaviruses

Zhenghao Yu, Mouraya Hussein, Yuanling Bao, Ana Alcalá-Lalinde, Pascal Zion Kroon, Eva Thuillier, Zhi Zhang, Luis Enjuanes, Sonia Zuñiga, Ben Berkhout, and Elena Herrera-Carrillo

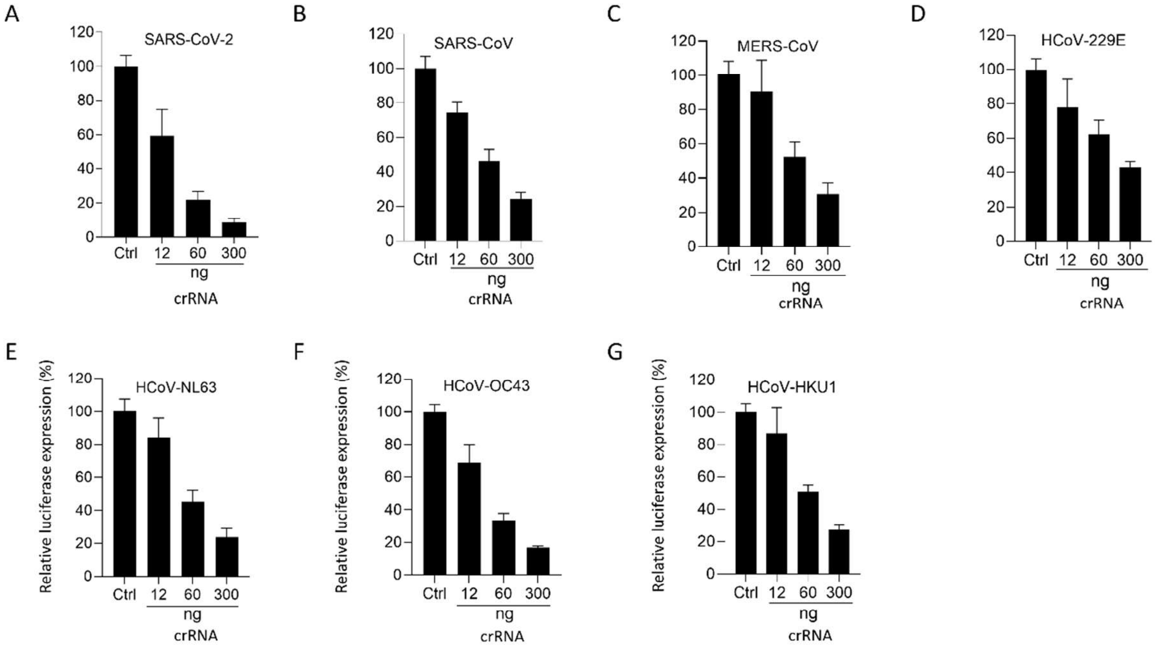

Figure S1. Dose-dependent inhibition of human coronaviruses by CRISPR-Cas13d/crRNA. The effect of Cas13d/crRNA2 dose on viral gene expression was evaluated using three transfection doses (12 ng, 60 ng, and 300 ng). After two days, luciferase levels were measured to assess the inhibition efficiency. A non-targeting crRNA (Ctrl), which does not target any human coronavirus, served as a negative control, with its luciferase expression normalized to 100%. Luciferase levels at different transfection doses are shown as percentages relative to the control (Ctrl).

Table S1. List of sequences inserted into the luciferase reporter

Human coronavirus	Sequence(5’-3’)
SARS-CoV-2	gtttatagtgatgtagaaaaccctcaccttATGGGTTGGGATTATCCTAAATGTGAtagagccatgcctaacatgcttagaattat
SARS-CoV	gtttacagtgatgtagaaactccacaccttATGGGTTGGGATTATCCAAAATGTGAcagagccatgcctaacatgcttaggataat
MERS-CoV	ttgtataaagatgttgataatccgcatcttATGGGTTGGGATTACCCTAAGTGTGAtagagctatgcctaatatgtgtagaatctt
HCoV-229E	ctgatggccgatgttgacgatcctaaattgATGGGATGGGACTATCCTAAGTGTGAtagagctatgccctcaatgattcgtatgtt
HCoV-NL63	ttaatcgatggtgttgaaaaccctatgctcATGGGTTGGGATTATCCCAAATGTGAtagagctttgcctaacatgatacgtatgat
HCoV-OC43	cttattaaagatgtcgacaatcctgtacttATGGGTTGGGATTATCCTAAGTGTGAtcgtgctatgccaaacatactacgtattgt
HCoV-HKU1	cttataaaggatgttgacaaccctgttcttATGGGTTGGGATTATCCTAAATGTGAtcgtgctatgccaaatattttgcgtattgt

Uppercase letters in the sequences indicate the target regions of each human coronavirus, while lowercase letters represent the 30-nucleotide flanking regions derived from the corresponding viral genome.

Table S2. Primers and probes used for qPCR and detection of escape virus.

Name	Sequence (5'-3')
SARS-CoV-2-sgmRNA-Forward	CCAACCAACTTTCGATCTCTTGT
SARS-CoV-2-sgmRNA-Reverse	GGGTGCATTTCGCTGATTTT
SARS-CoV-2-sgmRNA-Probe	TTCTCTAAACGAACAAACTA
SARS-CoV-sgmRNA-Forward	GCCAACCAACCTCGATCTCTTGT
SARS-CoV-sgmRNA-Reverse	AATCTGTGGGTCCACCAAATGTA
SARS-CoV-sgmRNA-Probe	CTCTAAACGAACAAATTA
MERS-CoV-sgmRNA-Forward	CTTCCCCTCGTTCTCTTGCA
MERS-CoV-sgmRNA-Reverse	TCATTGTTATCGGCAAAGGAAA
MERS-CoV-sgmRNA-Probe	CTTTGATTTTAACGAATCTC
HCov-229E-sgmRNA-Forward	CAGATAGAAAAGTTGCTTTTTAGAC
HCov-229E-sgmRNA-Reverse	CAGATGCATCAGCCCATTTGAC
HCov-229E-sgmRNA-Probe	TACTTTTCTAAACTGAACGA
HCov-NL63-sgmRNA-Forward	ATAGATAGAGAATTTTCTTATTTAGAC
HCov-NL63-sgmRNA-Reverse	AATTTCTTCCTAGCAGCTCTGT
HCov-NL63-sgmRNA-Probe	ACTAAACAAAATGGCTAGTG
HCov-229E-gRNA-Forward	GAACCTGATGGCCGATGTTG
HCov-229E-gRNA-Reverse	GCTTAGAACCTAAAATCATAGCC
HCov-229E-gRNA-Probe	AAATTGATGGGATGGGACTATCCTAAGTG
HCov-NL63-gRNA-Forward	GTTGCGTACTTTAATTGATGGTGT
HCov-NL63-gRNA-Reverse	AGAGCCCAACACCATGGCTG
HCov-NL63-gRNA-Probe	CTTATGGGTTGGGATTATCCCAAATGT
18s-Forward	CGGCTACCACATCCAAGGAA
18s-Reverse	GCTGGAATTACCGCGGCT
18s-Probe	TGCTGGCACCAGACTTGCCCTC
HCoV-229E-escape-Forward	GGAACGTGCACGTACAGTGGG
HCoV-229E-escape-Reverse	GGCGTAGGCTGTAGTTGCATC
HCoV-NL63-escape-Forward	CACGCAATGCCACTGTTGTT
HCoV-NL63-escape-Reverse	GCGTCACCAGAAGTCGTACC

Table S3. Primers used for the production of crRNAs.

Name	Sequence (5'-3')
crRNA2-F	TAATACGACTCACTATAGGGaacccctaccaactggtcggggtttgaaacCACATTTAGGATAATCCCAACCC
crRNA2-R	GGGTTGGGATTATCCTAAATGTGgtttcaaaccccgaccagttggtaggggttCCCTATAGTGAGTCGTATTA
crRNA3-F	TAATACGACTCACTATAGGGaacccctaccaactggtcggggtttgaaacACATTTAGGATAATCCCAACCCA
crRNA3-R	TGGGTTGGGATTATCCTAAATGTgtttcaaaccccgaccagttggtaggggttCCCTATAGTGAGTCGTATTA
